# Supplementary material for: A gene expression signature of TREM2hi macrophages and γδ T cells predicts immunotherapy response
Source: Nat Commun. 2020 Oct 8;11:5084. doi: 10.1038/s41467-020-18546-x (PMC7545100; doi:10.1038/s41467-020-18546-x)
Supplement: Supplementary file 1 — Supplementary Information [file 41467_2020_18546_MOESM1_ESM.pdf]

Supplementary Information

A gene expression signature of TREM2^{hi} macrophages and γδ T cells predicts immunotherapy response.

Xiong et al.

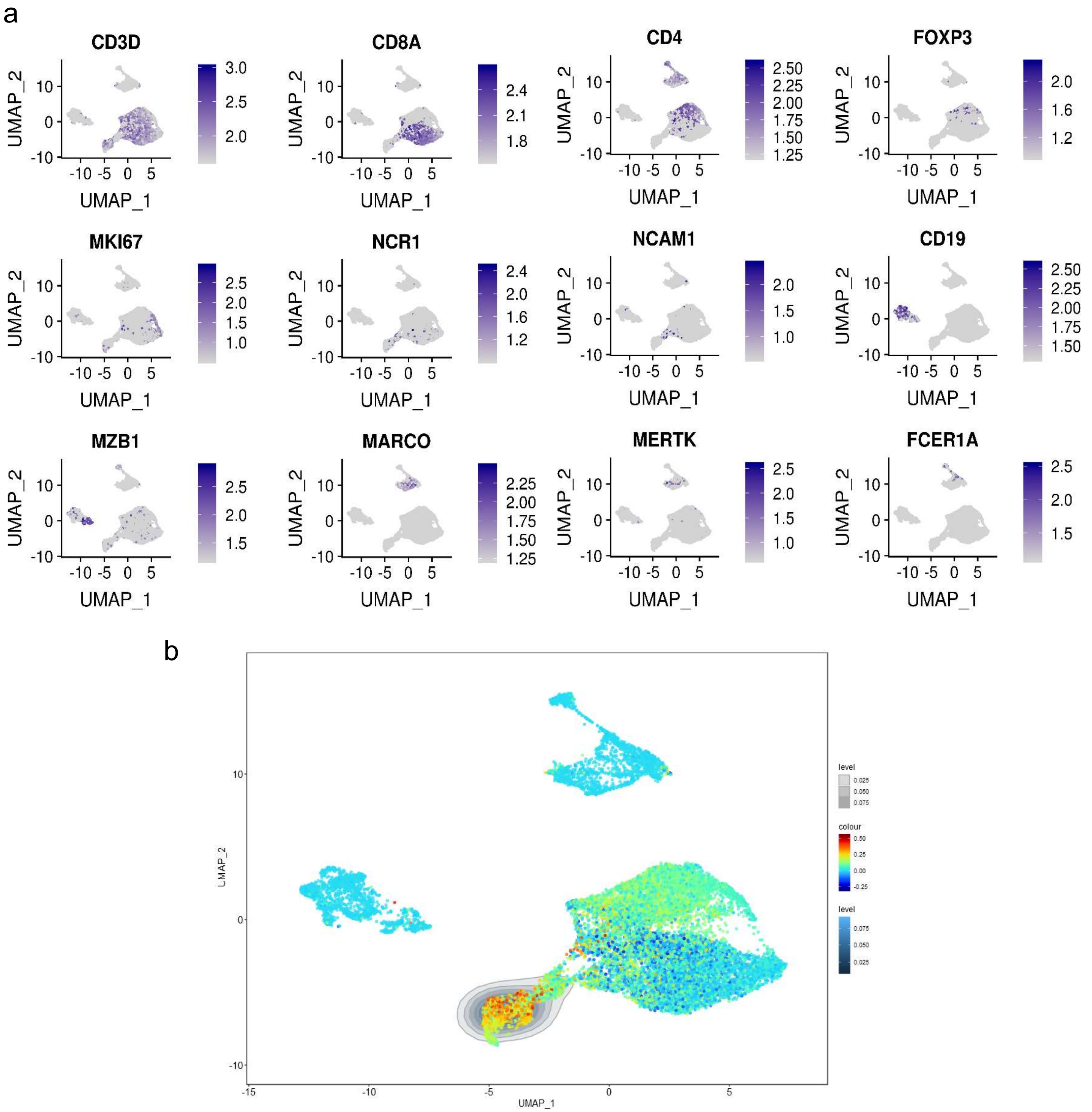

Supplementary Figure 1. The characterization of the ten major immune cell populations according to their respective canonical marker expression status. The scRNA-seq dataset - GSE120575 was used in this analysis. (a) The expression of canonical marker genes in different clusters of single cells; (b) Validation of our defined γδ T lymphocytes by the expression of the published gene expression signatures of γδ T cells.

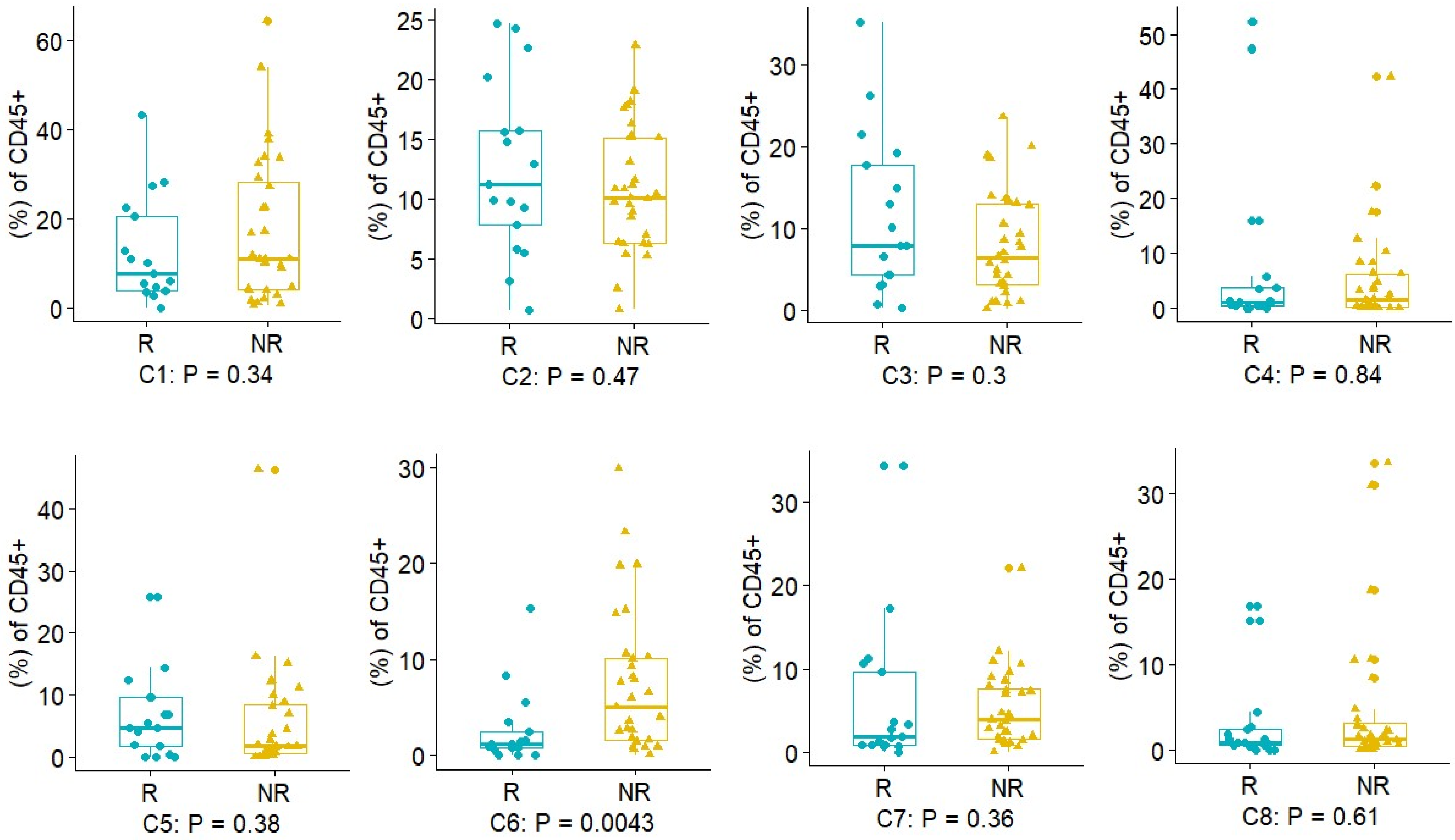
(%) of CD45+
R
NR
C1: P = 0.34
C2: P = 0.47
C3: P = 0.3
C4: P = 0.84
C5: P = 0.38
C6: P = 0.0043
C7: P = 0.36
C8: P = 0.61

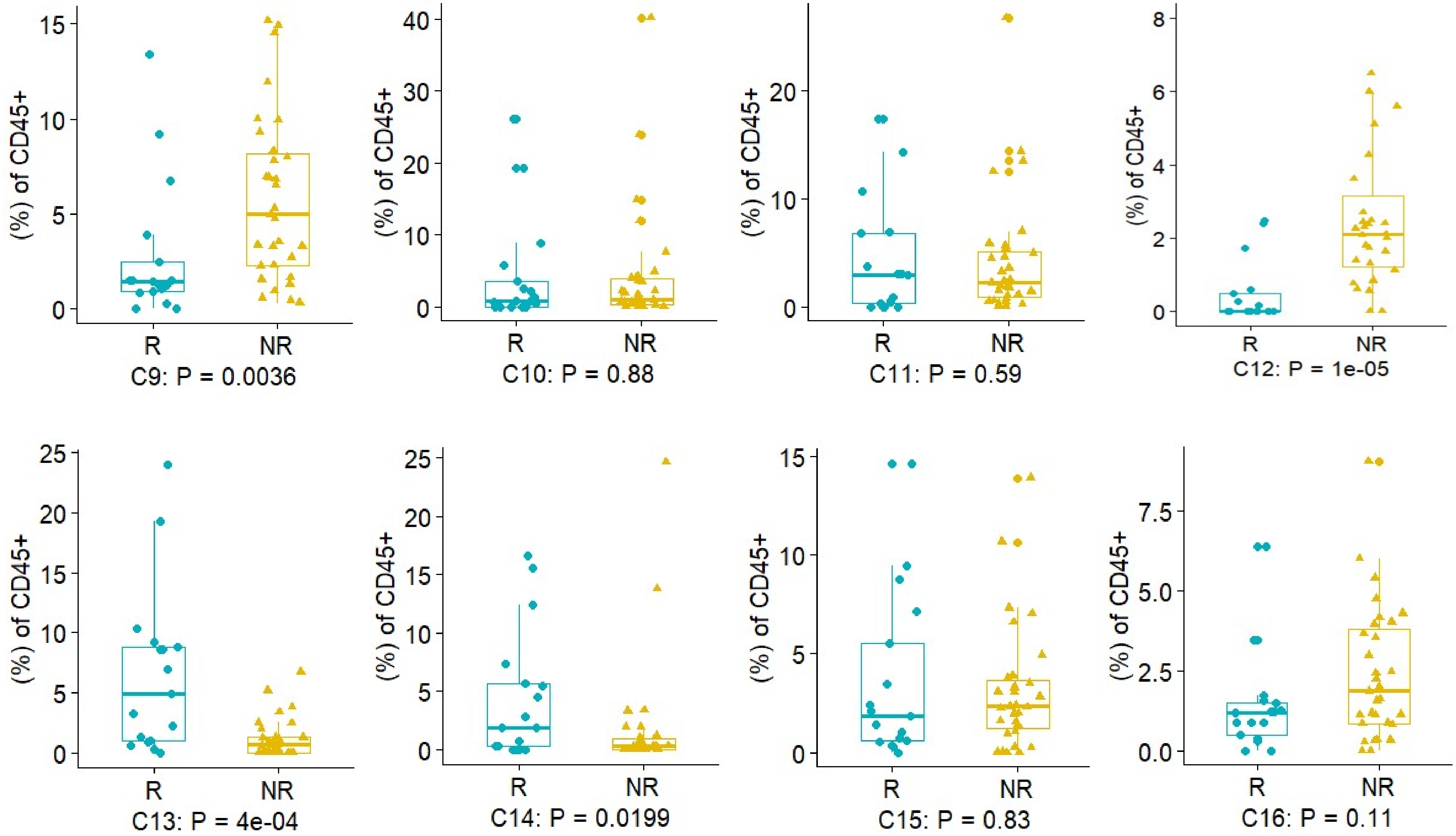

(%) of CD45+
R
NR
C9: P = 0.0036
C10: P = 0.88
C11: P = 0.59
C12: P = 1e-05
C13: P = 4e-04
C14: P = 0.0199
C15: P = 0.83
C16: P = 0.11

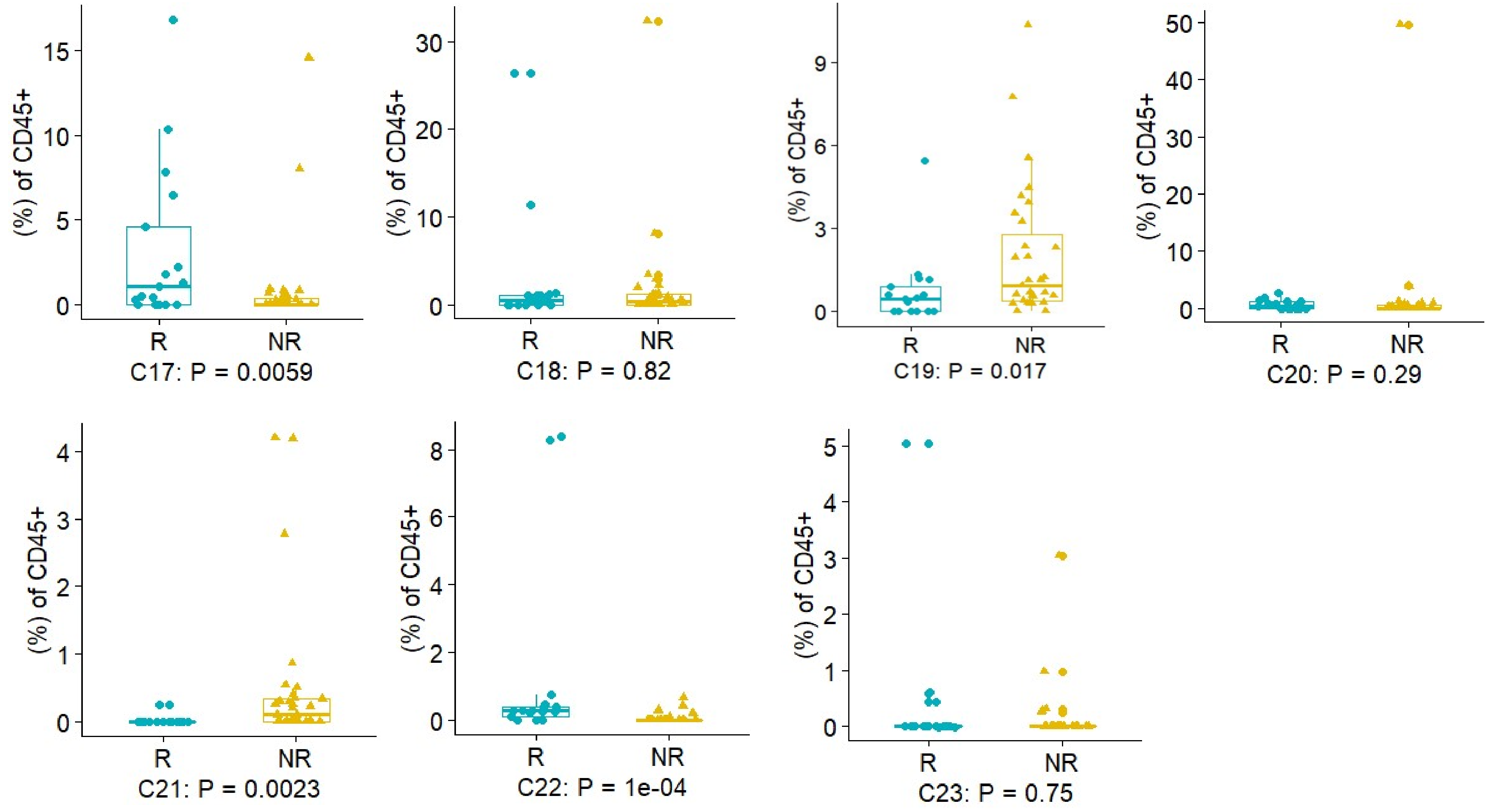

Supplementary Figure 2. Comparison of the abundance of each immune cell subset between the immune checkpoint therapy responder and non-responder groups. The scRNA-seq dataset - GSE120575 was used in this analysis. Boxplots showing the results of the Wilcoxon tests at the patient level for each of the 23 immune cell clusters were presented. There were 17 samples for the responder group and 31 samples for the non-responder group. Center line, median. Box limits, upper and lower quartiles. Whiskers, 1.5 interquartile range. Points beyond whiskers, outliers. The two-sided Wilcoxon tests were performed with no adjustment for multiple comparisons.

a

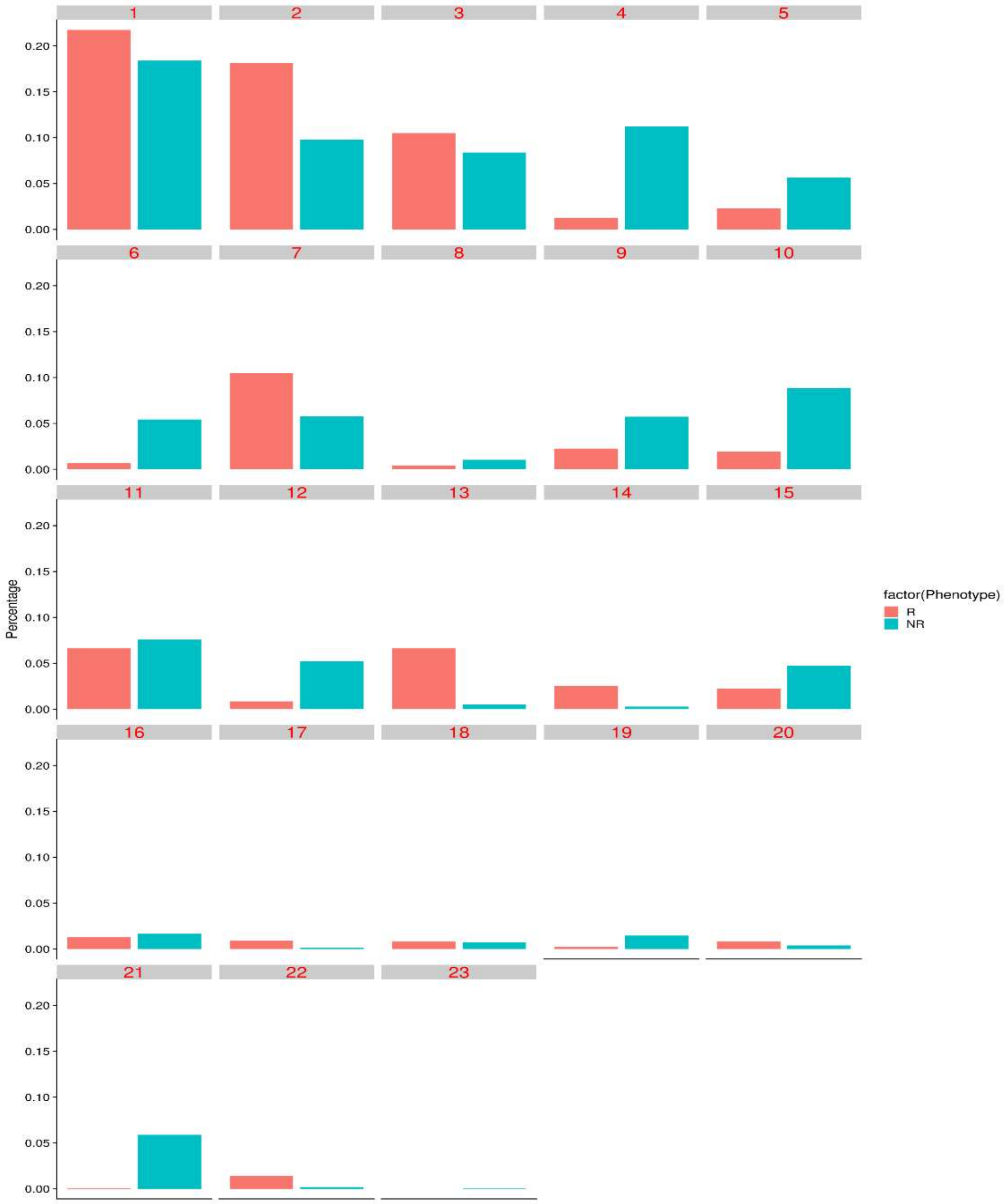
1
2
3
4
5
6
7
8
9
10
11
12
13
14
15
16
17
18
19
20
21
22
23
0.20
0.15
0.10
0.05
0.00
Percentage
factor(Phenotype)
R
NR

b

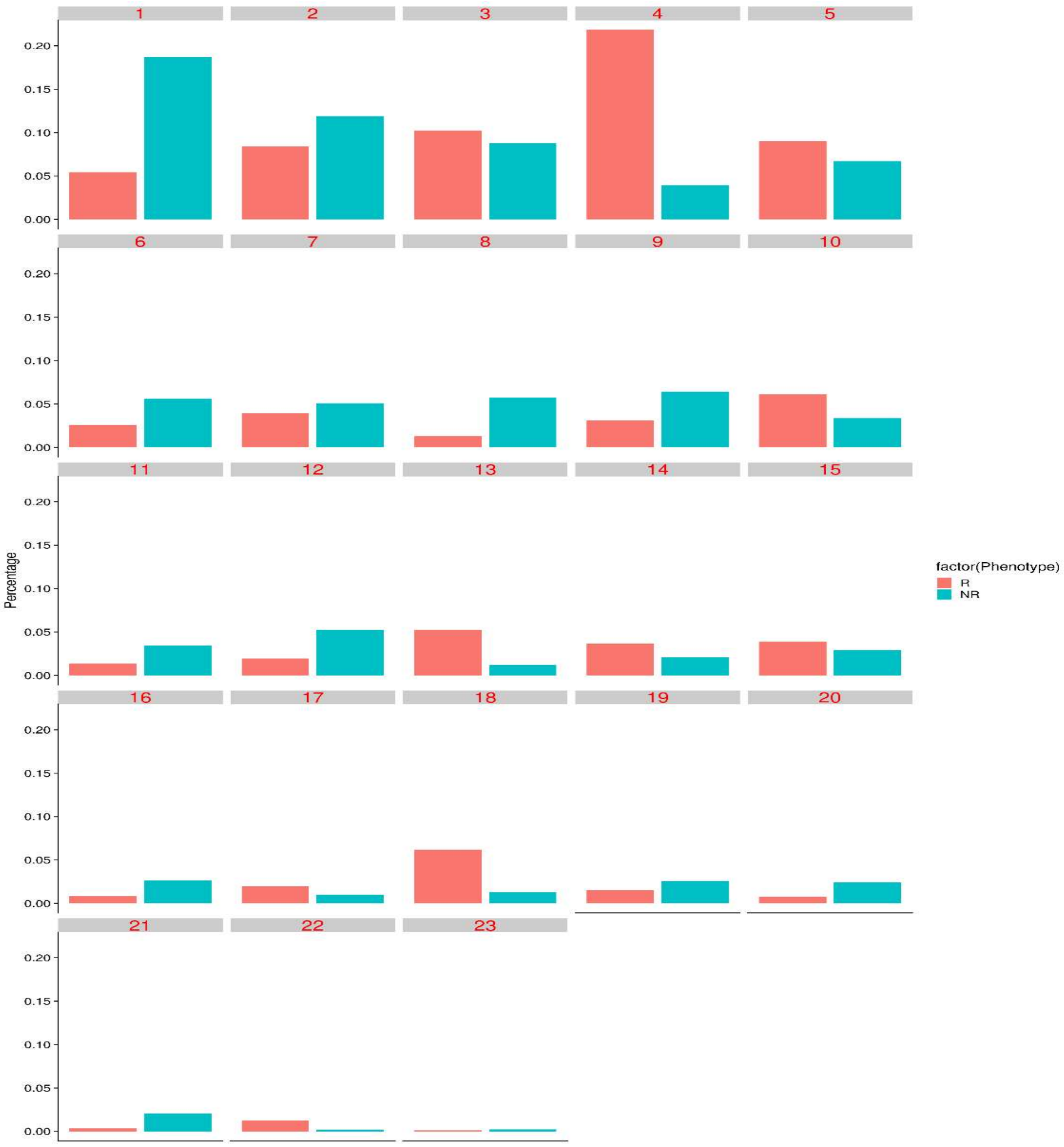
1
2
3
4
5
6
7
8
9
10
11
12
13
14
15
16
17
18
19
20
21
22
23
0.20
0.15
0.10
0.05
0.00
Percentage
factor(Phenotype)
R
NR

c

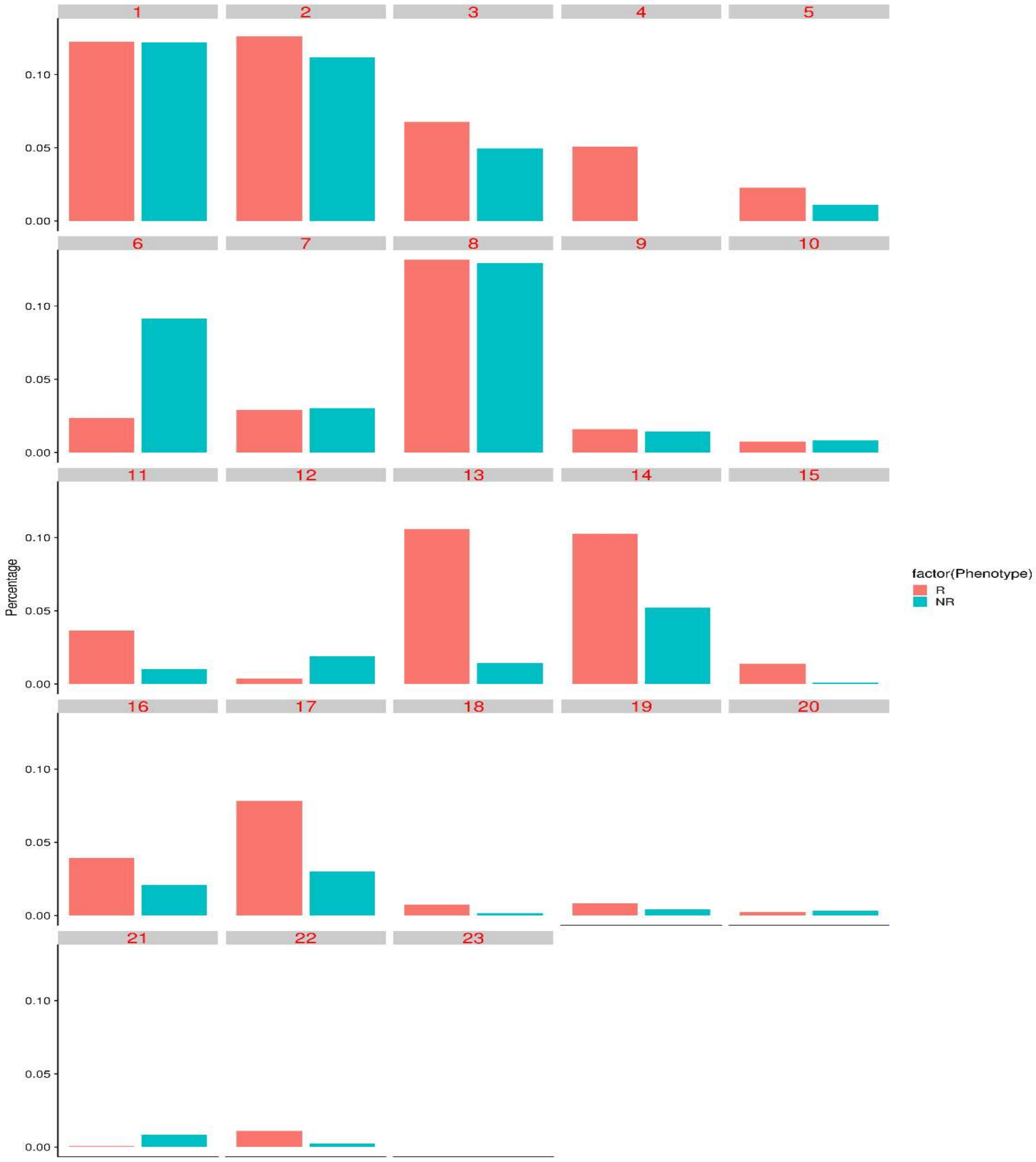

Supplementary Figure 3. Cell abundance comparison stratified by treatment schemes. The scRNA-seq dataset - GSE120575 was used in this analysis. The percentages (% of $CD45^{+}$ cells) of each of the 23 single-cell clusters for the responders (R) and non-responders (NR) groups of melanoma samples collected in the three scenarios. (a) before anti-PD-1 treatment; (b) after anti-PD-1 treatment; (c) after anti-CTLA4+anti-PD-1 treatment. No enough single cells were available for comparision between responders and non-responders for other scenarios. For the after anti-PD-1 treatment melanoma samples, the denominators for the R and the NR groups are 1524 and 6334, respectively; for the after anti-CTLA4 plus anti-PD-1 treatment melanoma samples, the denominators for the R and the NR groups are 1315 and 1190, respectively.

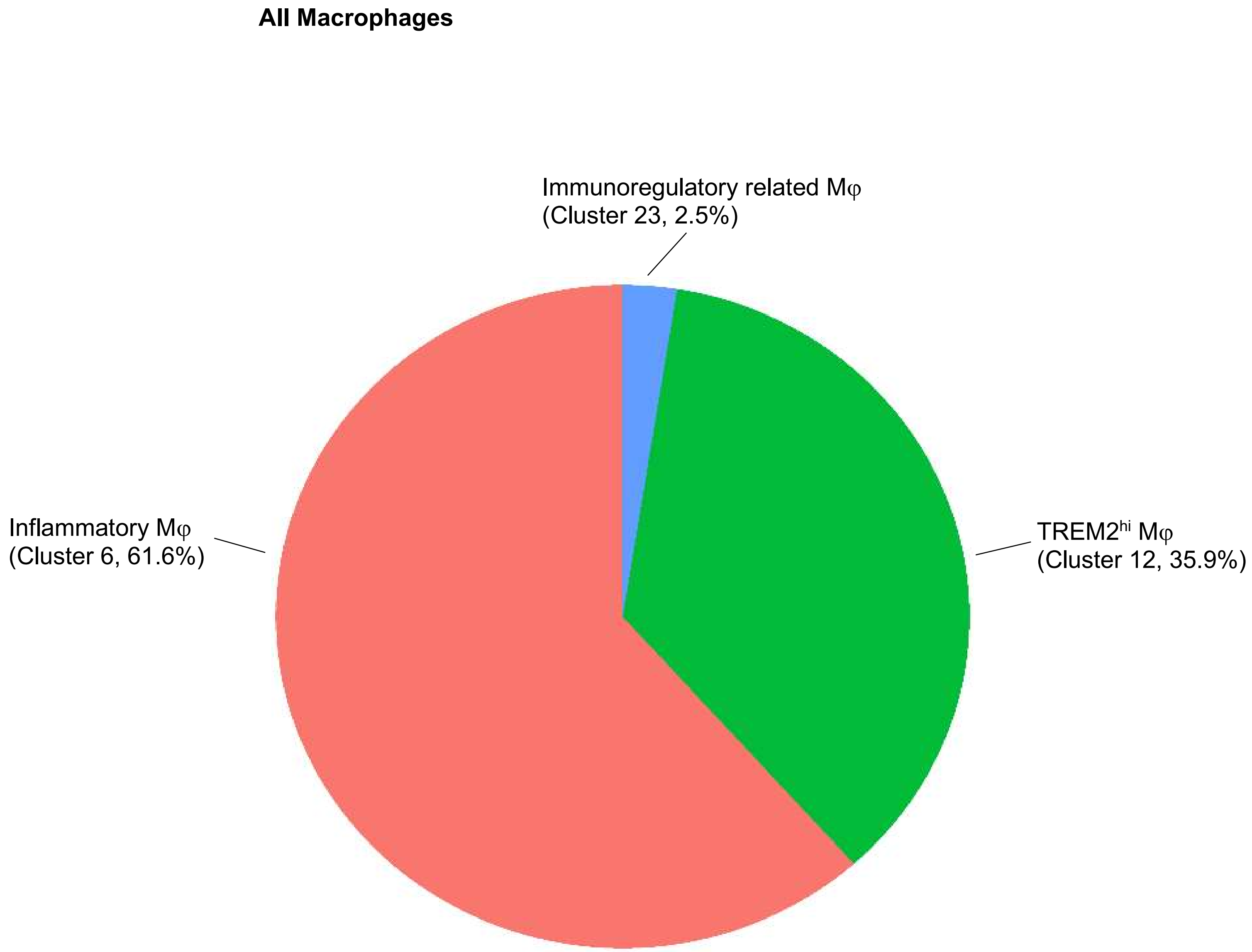

Supplementary Figure 4. Fraction of each macrophage subsets. The scRNA-seq dataset - GSE120575 was used in this analysis. Proportions of inflammatory macrophages (cluster 6), TREM2^{hi} macrophages (cluster 12), and Immunoregulatory related macrophages (cluster 23), the three macrophage subsets in immune cells from the melanoma tumor samples.

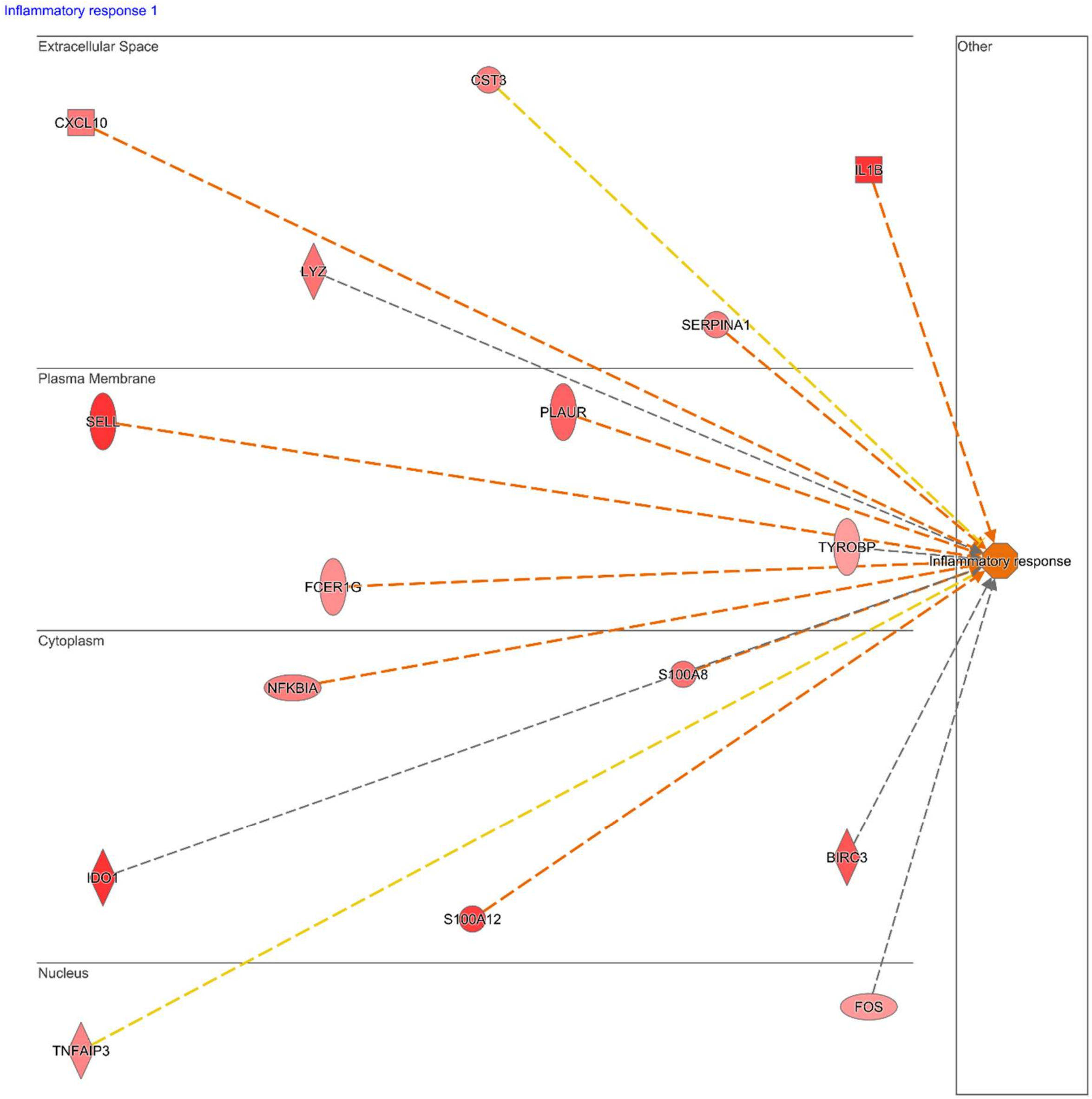

Supplementary Figure 5. Pathway analysis for macrophage cluster 6. The scRNA-seq dataset - GSE120575 was used in this analysis. IPA analyses based on the list of differentially expressed genes between cluster 6 and other macrophages. The results revealed that inflammatory response was significantly activated with a large number of overexpressed inflammatory marker genes in cluster 6 macrophages (adjusted P = 3.93E-10, activation Z score = 2.01). Cluster 6 macrophages population was thus identified as the ‘Inflammatory Mφ’.

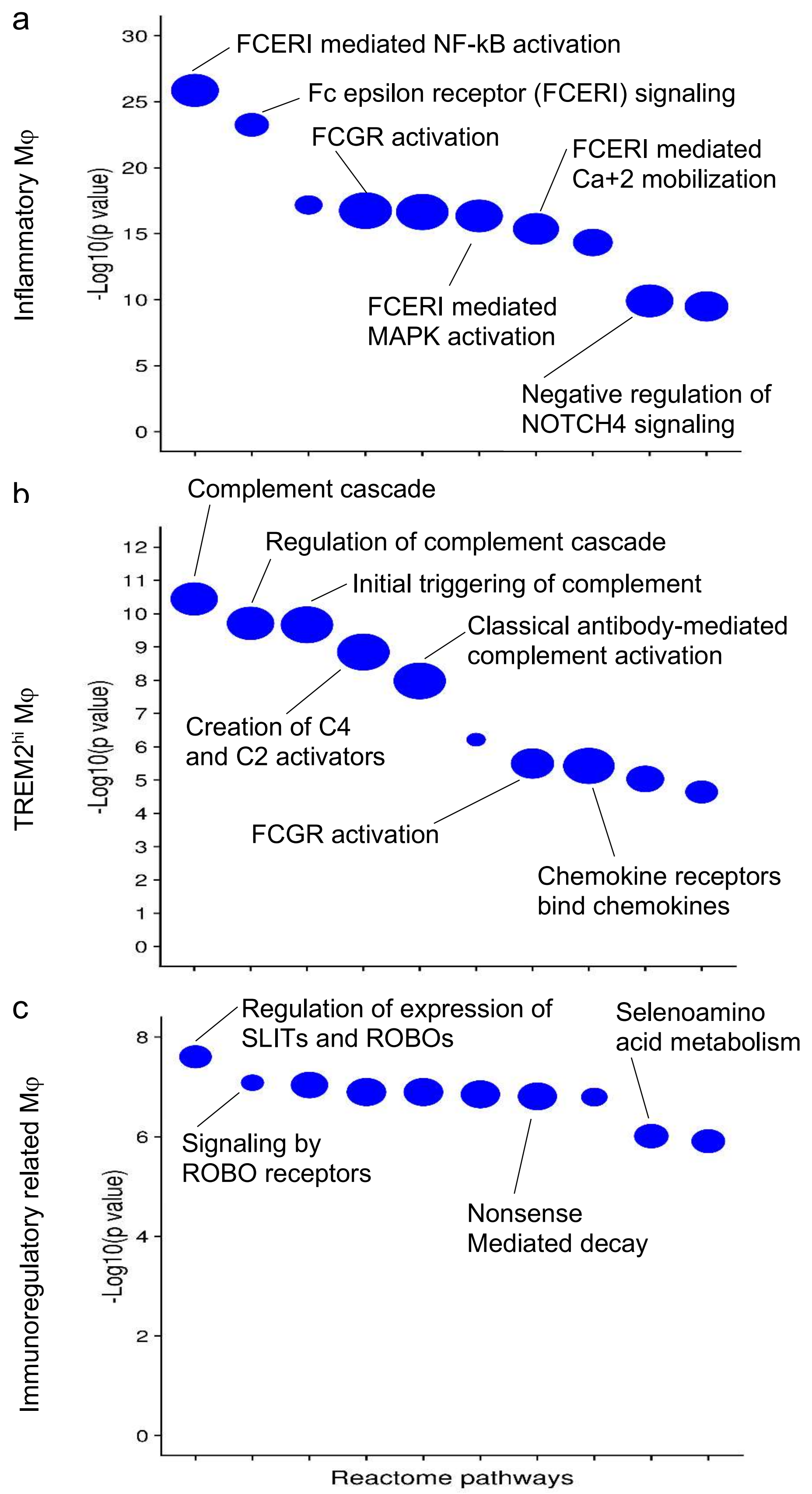

Supplementary Figure 6. Gene ontology enrichment analysis of three macrophages subsets. The scRNA-seq dataset - GSE120575 was used in this analysis. Gene ontology enrichment analysis of reactome pathways in (a) Inflammatory macrophages, (b) TREM2^{hi} macrophages, and (c) Immunoregulatory related macrophages infiltrating the melanoma tumor samples from patients subjected to ICT. Size of the circles is proportional to the fold difference.

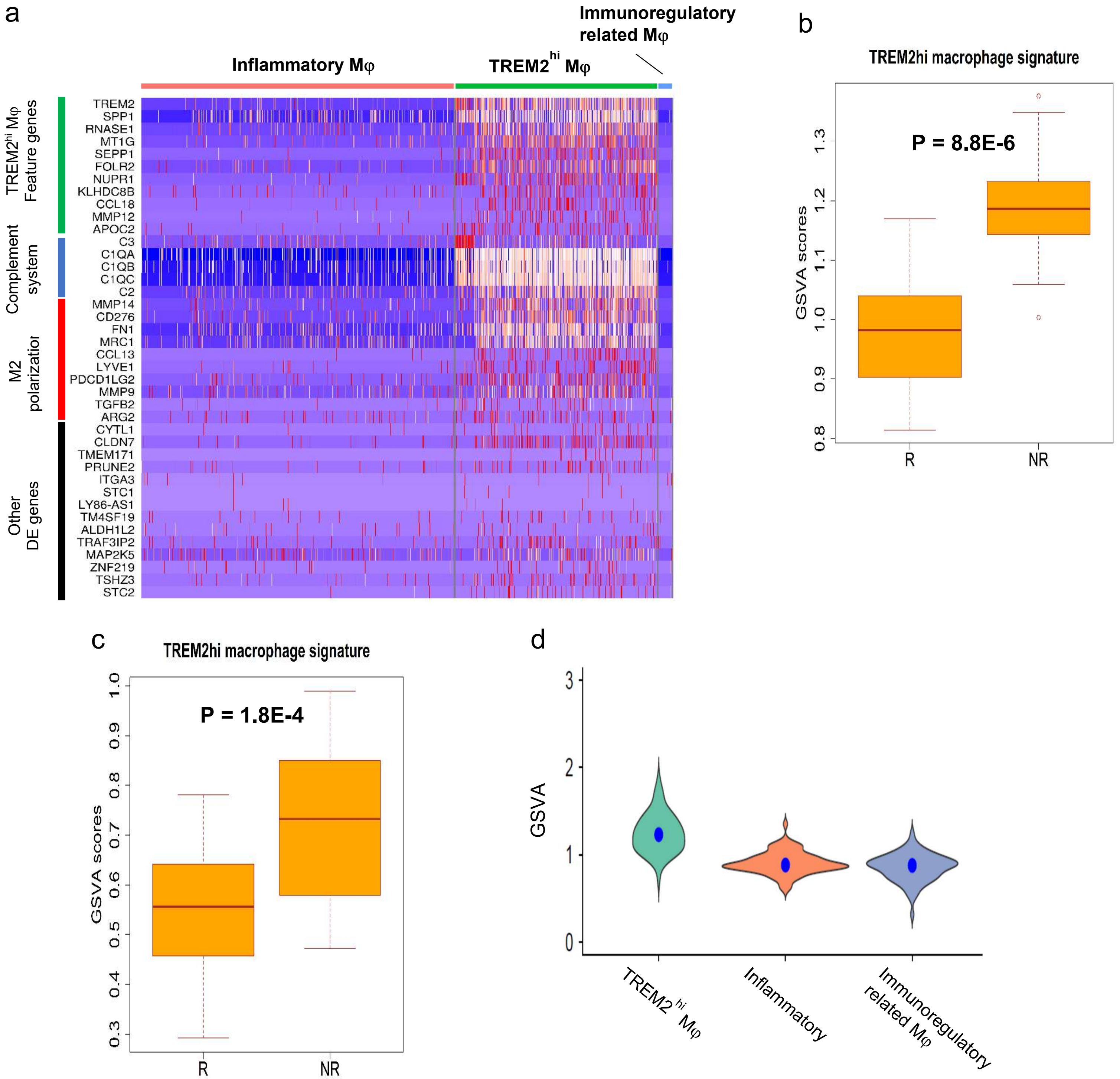

Supplementary Figure 7. A 40-gene expression signature that can characterize the TREM2hi macrophage population. (a) The scRNA-seq dataset - GSE120575 was analyzed, which generated the heatmap of the expression of a 40-gene signature representing the TREM2hi macrophage population. In the boxplots for (b) GSE78220 dataset and (c) GSE91061 dataset, the GSVA scores of the TREM2hi macrophage geneset were significantly higher in the ICT non-responding tumors than the responding tumors. Center line, median. Box limits, upper and lower quartiles. Whiskers, 1.5 interquartile range. Points beyond whiskers, outliers. For (b) and (c), the two-sided t-tests were performed with no adjustment for multiple comparisons. (d) Violin plot showed that the actitiy of this gene set is higher in the TREM2hi macrophages compared to the other macrophages in the GSE120575 dataset.

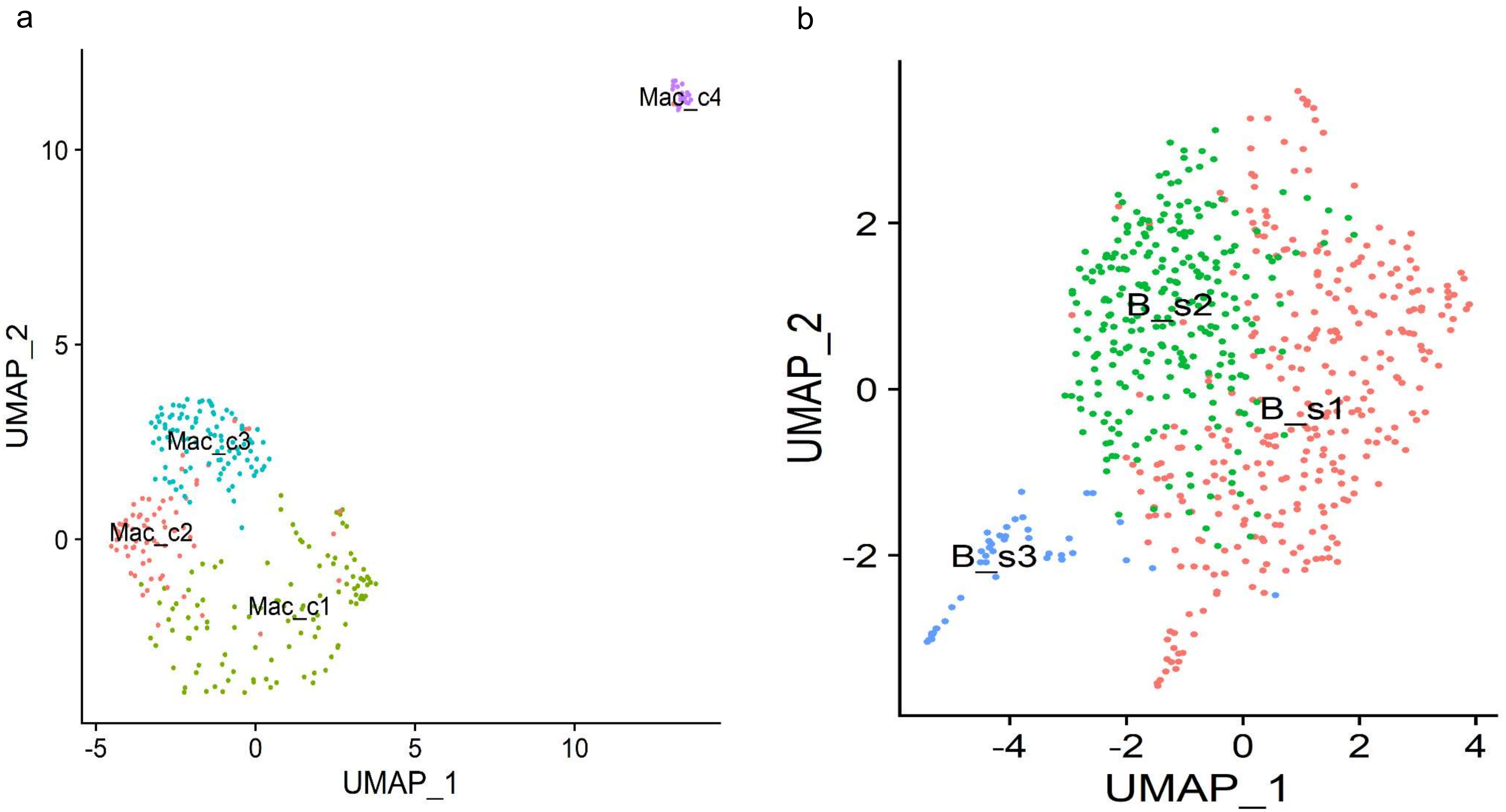

a
Mac_c4
Mac_c3
Mac_c2
Mac_c1
UMAP_2
UMAP_1
10
5
0
-5
0
5
10
b
B_s2
B_s1
B_s3
UMAP_2
UMAP_1
2
0
-2
-4
-2
0
2
4

c

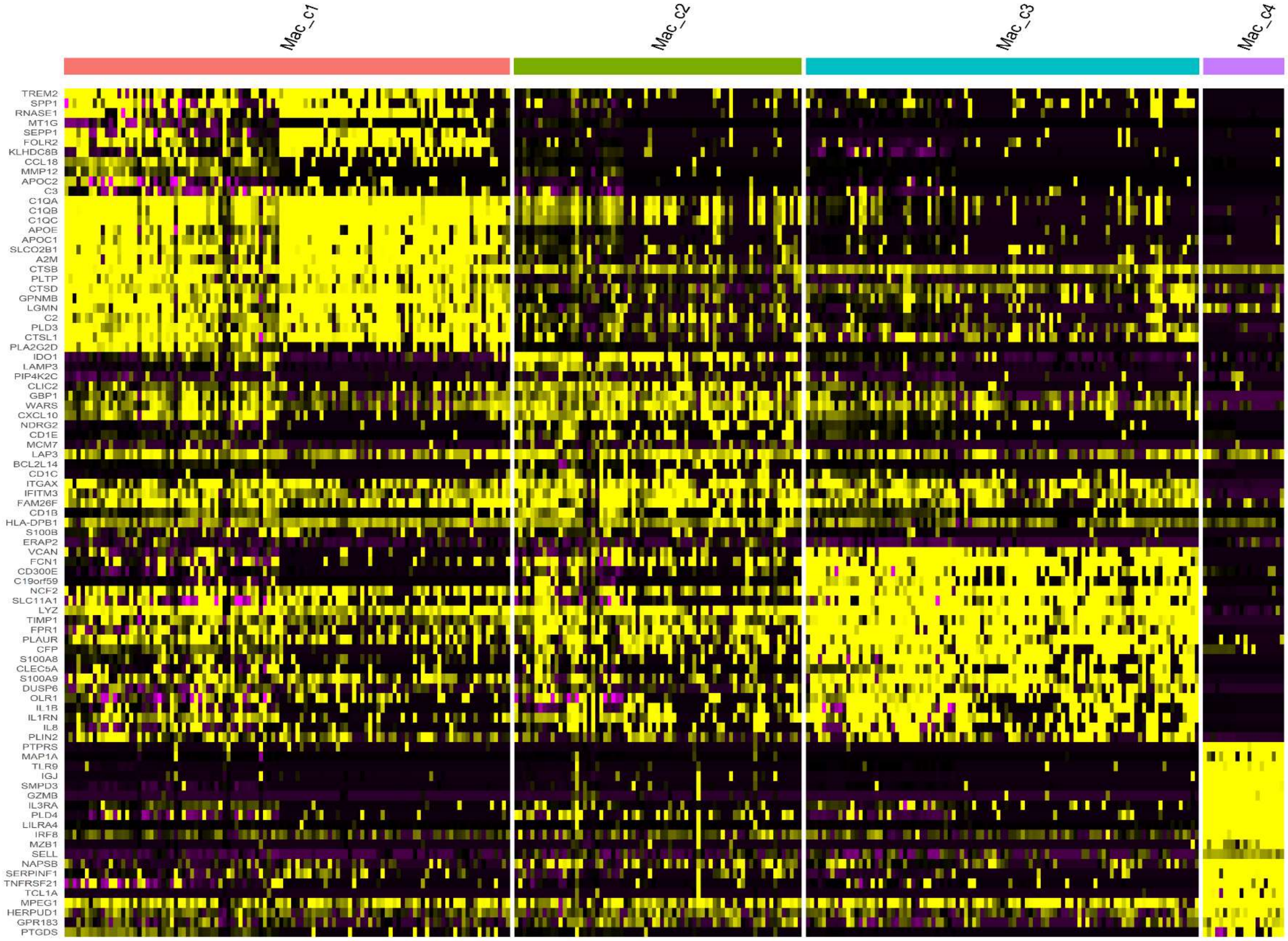

Mac_c1
Mac_c2
Mac_c3
Mac_c4
TREM2
SPP1
RNASE1
MT1G
SEPP1
FOLR2
KLHDC8B
CCL18
MMP12
APOC2
C3
C1QA
C1QB
C1QC
APOE
APOC1
SLCO2B1
A2M
CTSB
PLTP
CTSD
GPNMB
LGMN
C2
PLD3
CTSL1
PLA2G2D
IDO1
LAMP3
PIP4K2C
CLIC2
GBP1
WARS
CXCL10
NDRG2
CD1E
MCM7
LAP3
BCL2L14
CD1C
ITGAX
IFITM3
FAM26F
CD1B
HLA-DPB1
S100B
ERAP2
VCAN
FCN1
CD300E
C19orf59
NCF2
SLC11A1
LYZ
TIMP1
FPR1
PLAUR
CFP
S100A8
CLEC5A
S100A9
DUSP6
OLR1
IL1B
IL1RN
IL8
PLIN2
PTPRS
MAP1A
TLR9
IGJ
SMPD3
GZMB
IL3RA
PLD4
LILRA4
IRF8
MZB1
SELL
NAPSB
SERPINF1
TNFRSF21
TCL1A
MPEG1
HERPUD1
GPR183
PTGDS

d

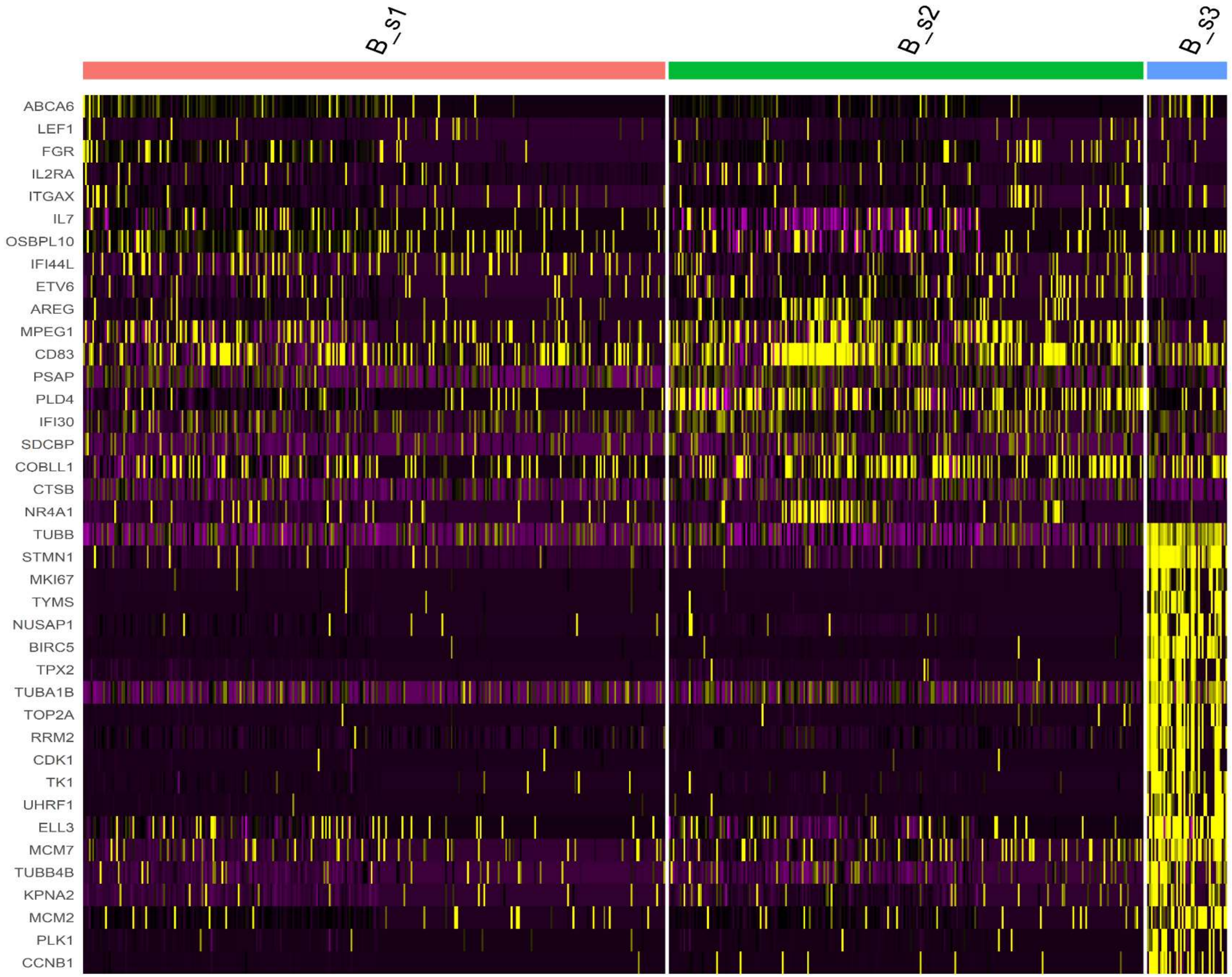
B_s1
B_s2
B_s3
ABCA6
LEF1
FGR
IL2RA
ITGAX
IL7
OSBPL10
IFI44L
ETV6
AREG
MPEG1
CD83
PSAP
PLD4
IFI30
SDCBP
COBLL1
CTSB
NR4A1
TUBB
STMN1
MKI67
TYMS
NUSAP1
BIRC5
TPX2
TUBA1B
TOP2A
RRM2
CDK1
TK1
UHRF1
ELL3
MCM7
TUBB4B
KPNA2
MCM2
PLK1
CCNB1

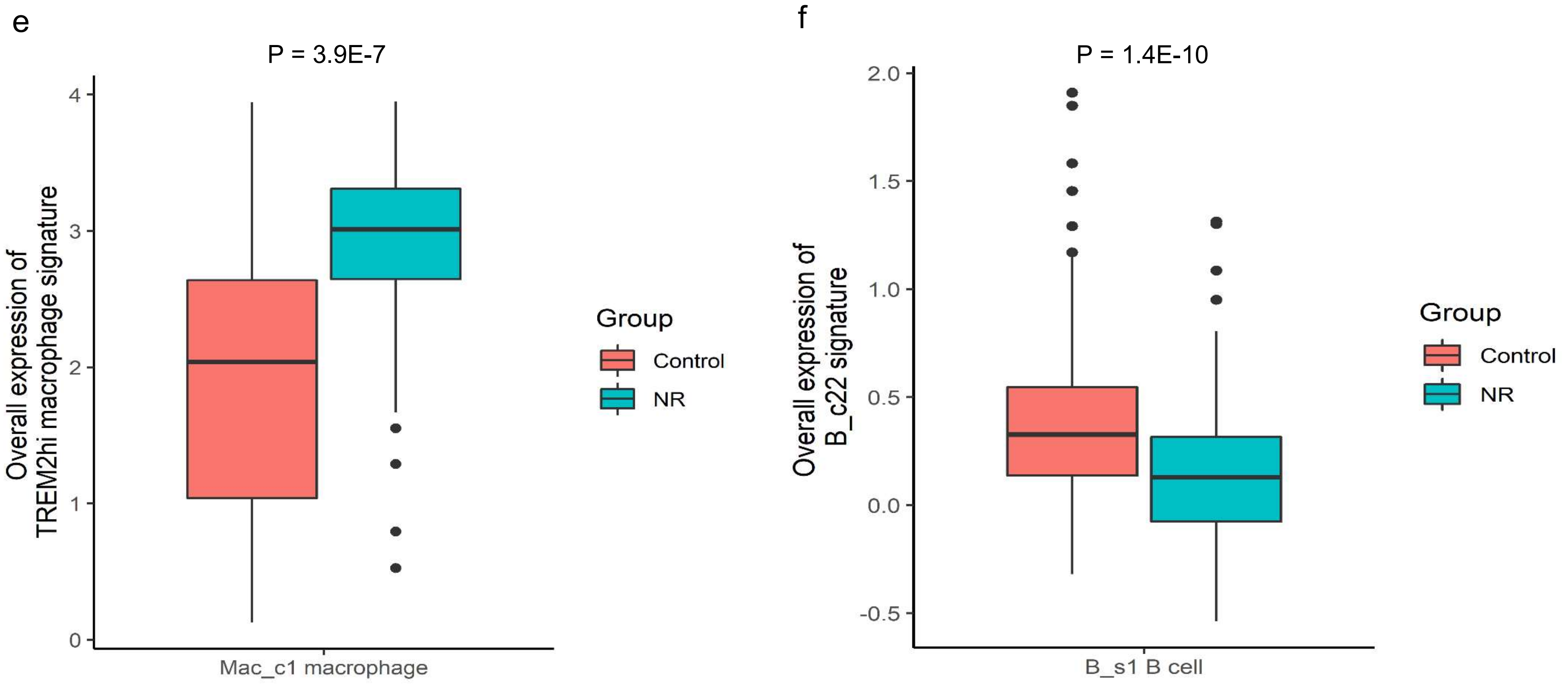

Supplementary Figure 8. Validation of the findings in melanoma using an independent scRNAseq dataset of melanoma - GSE115978. (a)-(b) The deeper clustering of the macrophages and B cells sequenced by Jerby-Arnon et al.'s scRNA-seq study showed the existence of the similar macrophage and B cell subpopulations that resemble our identified TREM2^{hi} macrophages and B_c22 B cells; (c) Heatmap of the four macrophage subclusters, which showed that the 'Mac_c1' macrophage subcluster overexpressed the TREM2^{hi} macrophage marker genes; (d) Heatmap of the three B cells subclusters, which showed that the 'B_s1' B cell subcluster overexpressed the B_c22 B cells marker genes; (e) the Mac_c1 macrophage subset had significantly higher overall expression of the TREM2^{hi} macrophage signature in the non-responders than the control samples; (f) The B_s1 B cell subset had significantly lower overall expression of the B_c22 B cell signature in the immunotherapy non-responders than the control samples. Center line, median. Box limits, upper and lower quartiles. Whiskers, 1.5 interquartile range. Points beyond whiskers, outliers. For (e) and (f), the two-sided Wilcoxon tests were performed with no adjustment for multiple comparisons.

a

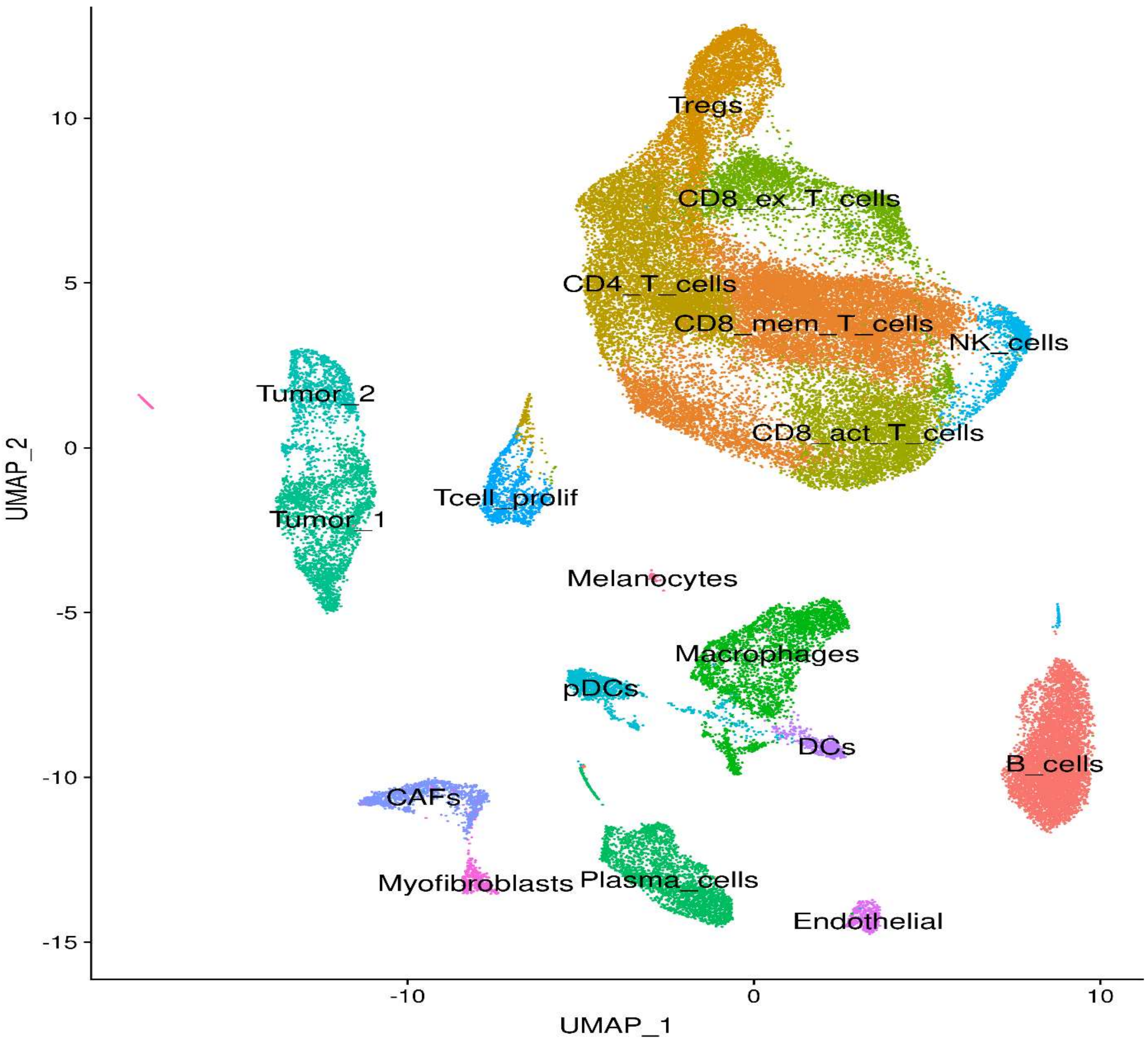
Tregs
CD8_ex_T_cells
CD4_T_cells
CD8_mem_T_cells
NK_cells
Tumor_2
CD8_act_T_cells
Tcell_prolif
Tumor_1
Melanocytes
Macrophages
pDCs
DCs
B_cells
CAFs
Myofibroblasts
Plasma_cells
Endothelial
10
5
0
-5
-10
-15
UMAP_2
-10
0
10
UMAP_1

b

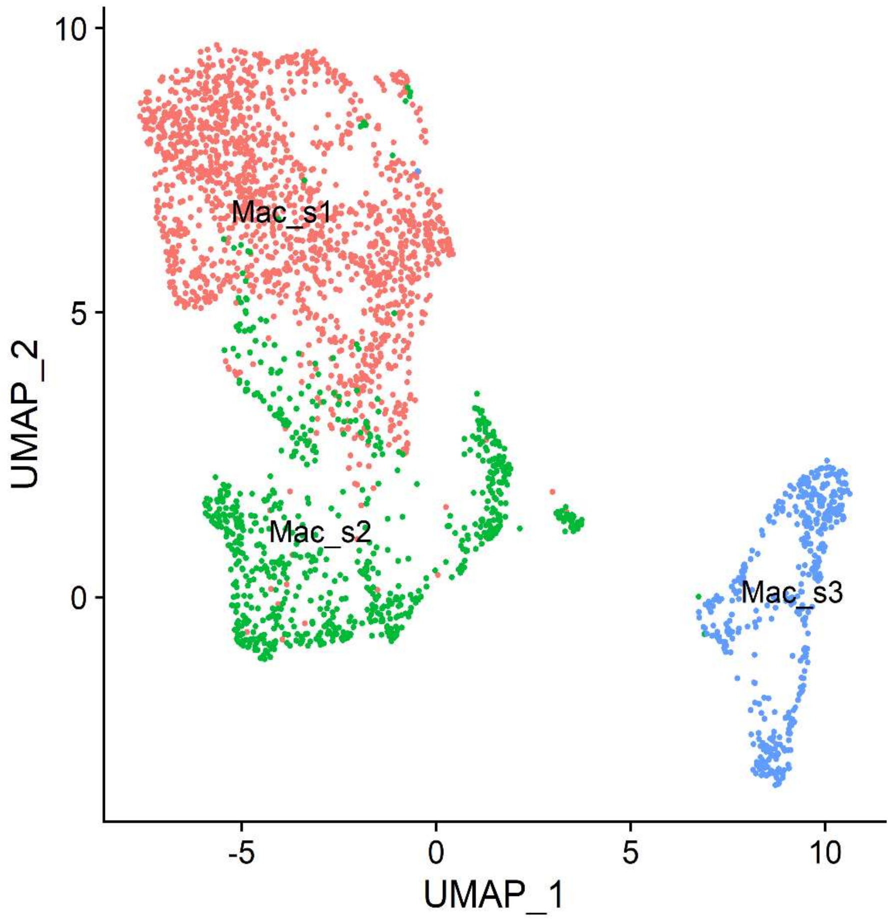
10
5
0
-5
0
5
10
UMAP_2
UMAP_1
Mac_s1
Mac_s2
Mac_s3

c

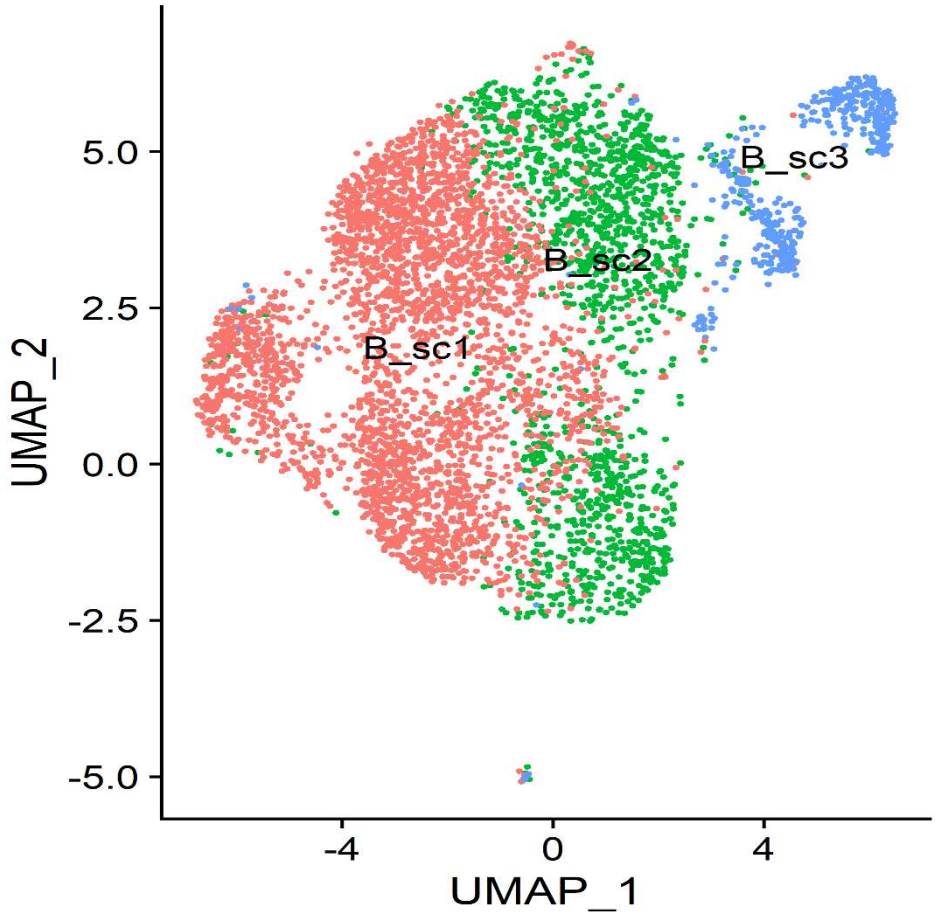
5.0
2.5
0.0
-2.5
-5.0
-4
0
4
UMAP_2
UMAP_1
B_sc1
B_sc2
B_sc3

d e

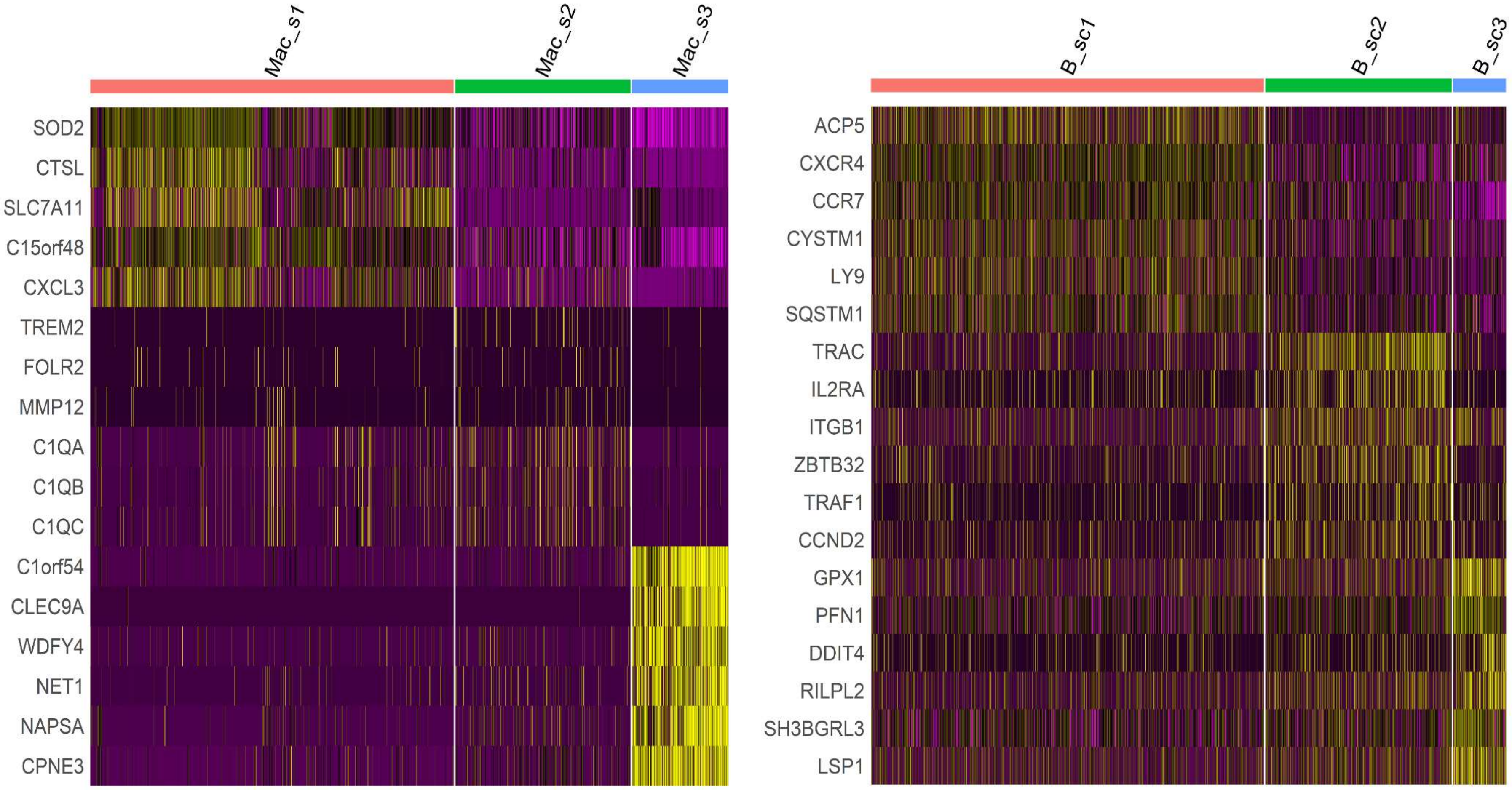
Mac_s1
Mac_s2
Mac_s3
SOD2
CTSL
SLC7A11
C15orf48
CXCL3
TREM2
FOLR2
MMP12
C1QA
C1QB
C1QC
C1orf54
CLEC9A
WDFY4
NET1
NAPSA
CPNE3
B_sc1
B_sc2
B_sc3
ACP5
CXCR4
CCR7
CYSTM1
LY9
SQSTM1
TRAC
IL2RA
ITGB1
ZBTB32
TRAF1
CCND2
GPX1
PFN1
DDIT4
RILPL2
SH3BGRL3
LSP1

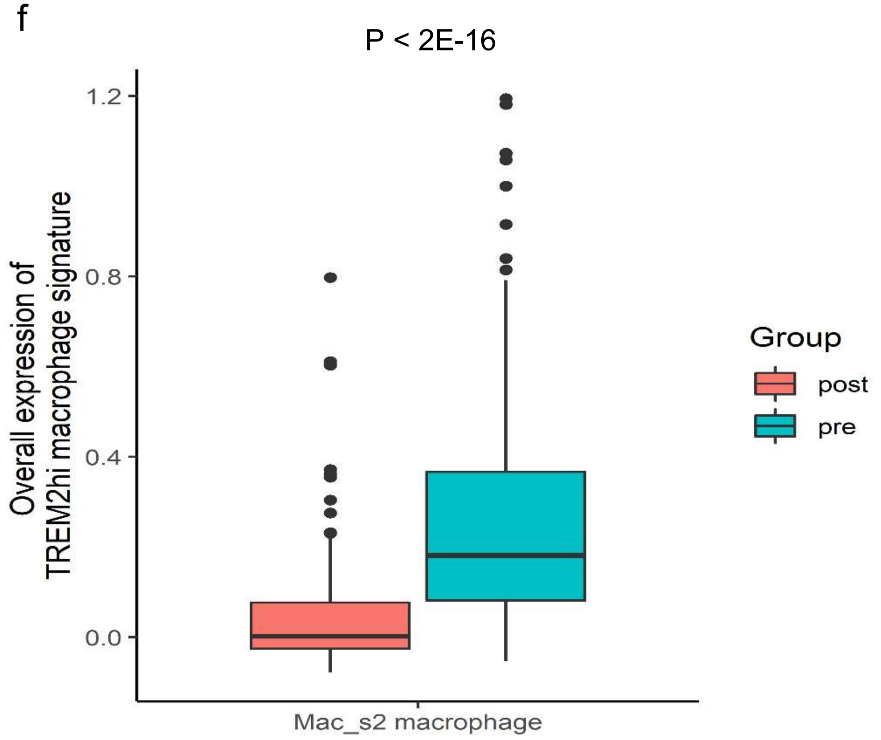

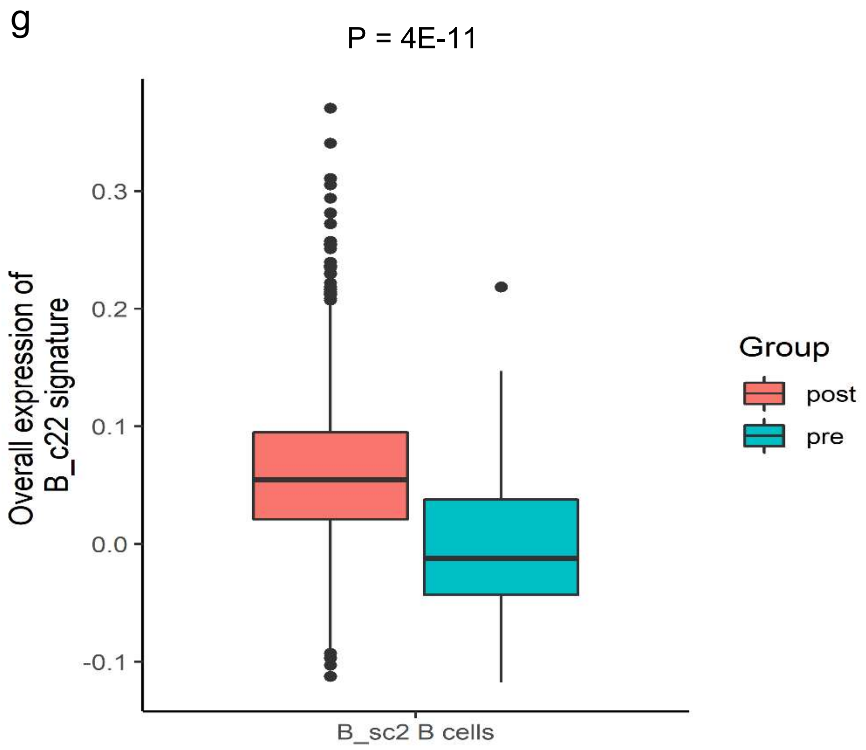

Supplementary Figure 9. Validation of the findings in melanoma using an scRNAseq dataset of basal cell carcinoma (BCC) - GSE123813.(a) General clustering analyses identified the overall macrophages and B cells populations; Finer clustering identified the macrophage (b) and B cell subpopulations (c) from the BCC tumors that are similar to the TREM2^{hi} macrophages and B_c22 B cells in the initial melanoma samples; (d) Heatmap showed that in the BCC dataset the 'Mac_s2' macrophage subcluster overexpressed the TREM2^{hi} macrophage marker genes; (e) Heatmap showed that 'B_sc2' B cell subcluster overexpressed the B_c22 B cells marker genes; (f) The Mac_s2 macrophage subset had significantly decreased overall expression of the TREM2^{hi} macrophage signature in the responsive BCC tumors after the anti-PD-1 therapy than the pretreatment BCC samples; (g) The B_sc2 B cell subset had significantly higher overall expression of the B_c22 signature in the post anti-PD-1 therapy responsive BCC tumors than the pretreatment BCC samples. Center line, median. Box limits, upper and lower quartiles. Whiskers, 1.5 interquartile range. Points beyond whiskers, outliers. For (f) and (g), the two-sided Wilcoxon tests were performed with no adjustment for multiple comparisons.

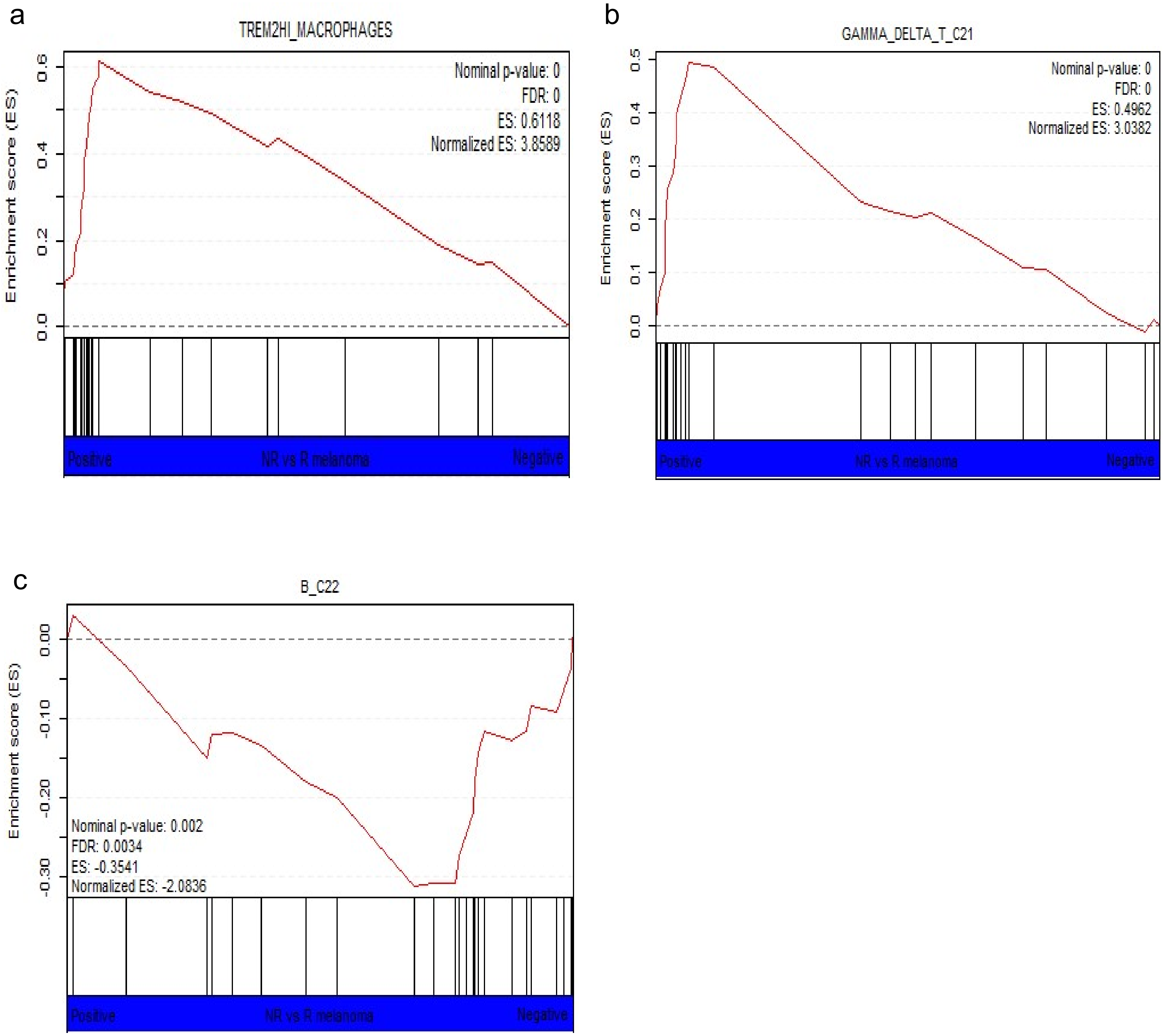

Supplementary Figure 10. The enrichment of the ImmuneCells.Sig signature for the characteristic genes of the immune cell subpopulations. The dataset - GSE78220 was used in this analysis. This ICT outcome signature was positively enriched for the characteristic genes of the (a) TREM2^{hi} Mφ, (b) Tgd_c21, and negatively enriched for the (c) B_c22.

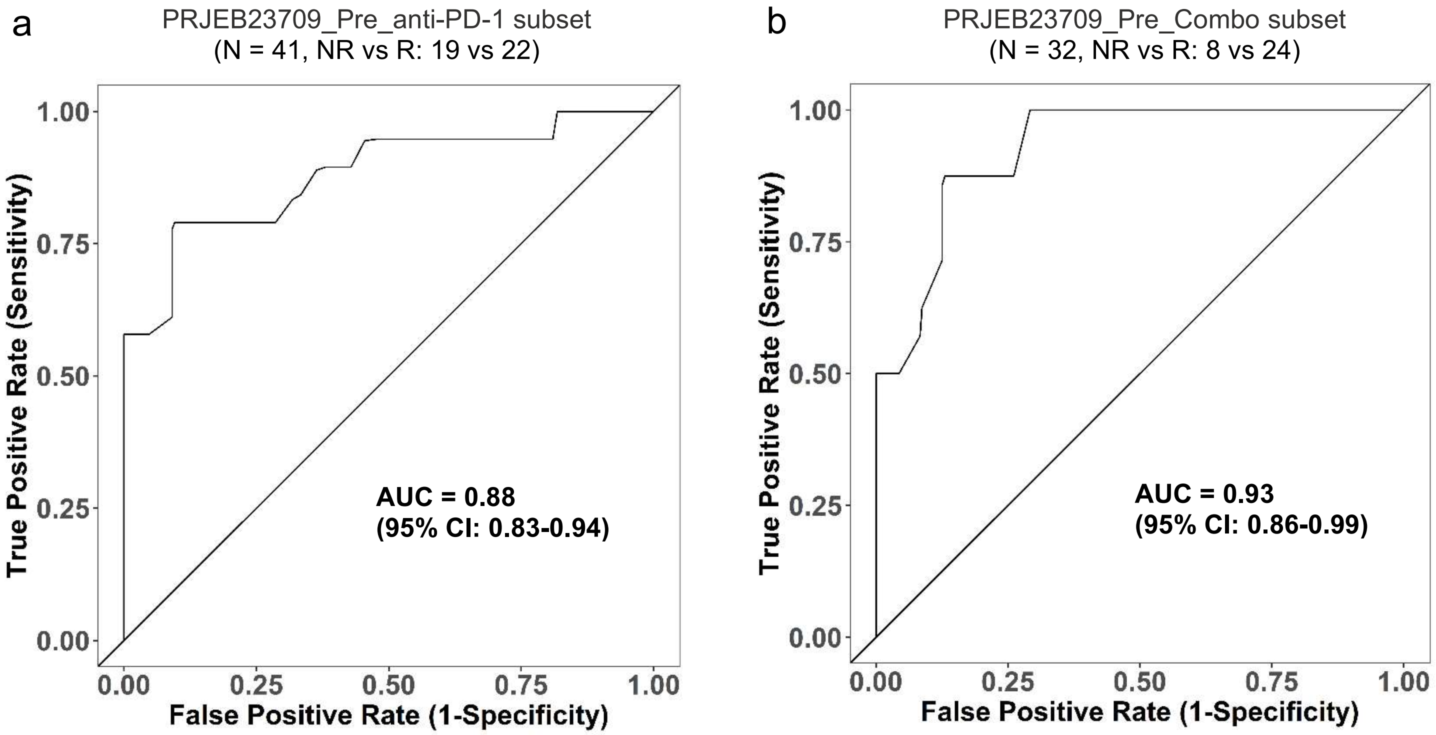

Supplementary Figure 11. Evaluation of the ImmuneCells.Sig signature. The dataset - PRJEB23709 was used in this analysis. The performance of the ImmuneCells.Sig signature in predicting ICT responders based on the pre-treatment melanoma biopies from patients subjected to different ICT regimen. ImmuneCells.Sig can accurately distinguish responders from non-responders in both Pre_anti-PD-1 and Pre_Combo subgroups (anti-PD-1 plus anti-CTLA-4) as can be seen in the ROC (receiver operating characteristic) curves of the (a) PRJEB23709_Pre_anti-PD-1 subset and (b) PRJEB23709_Pre_Combo subset.

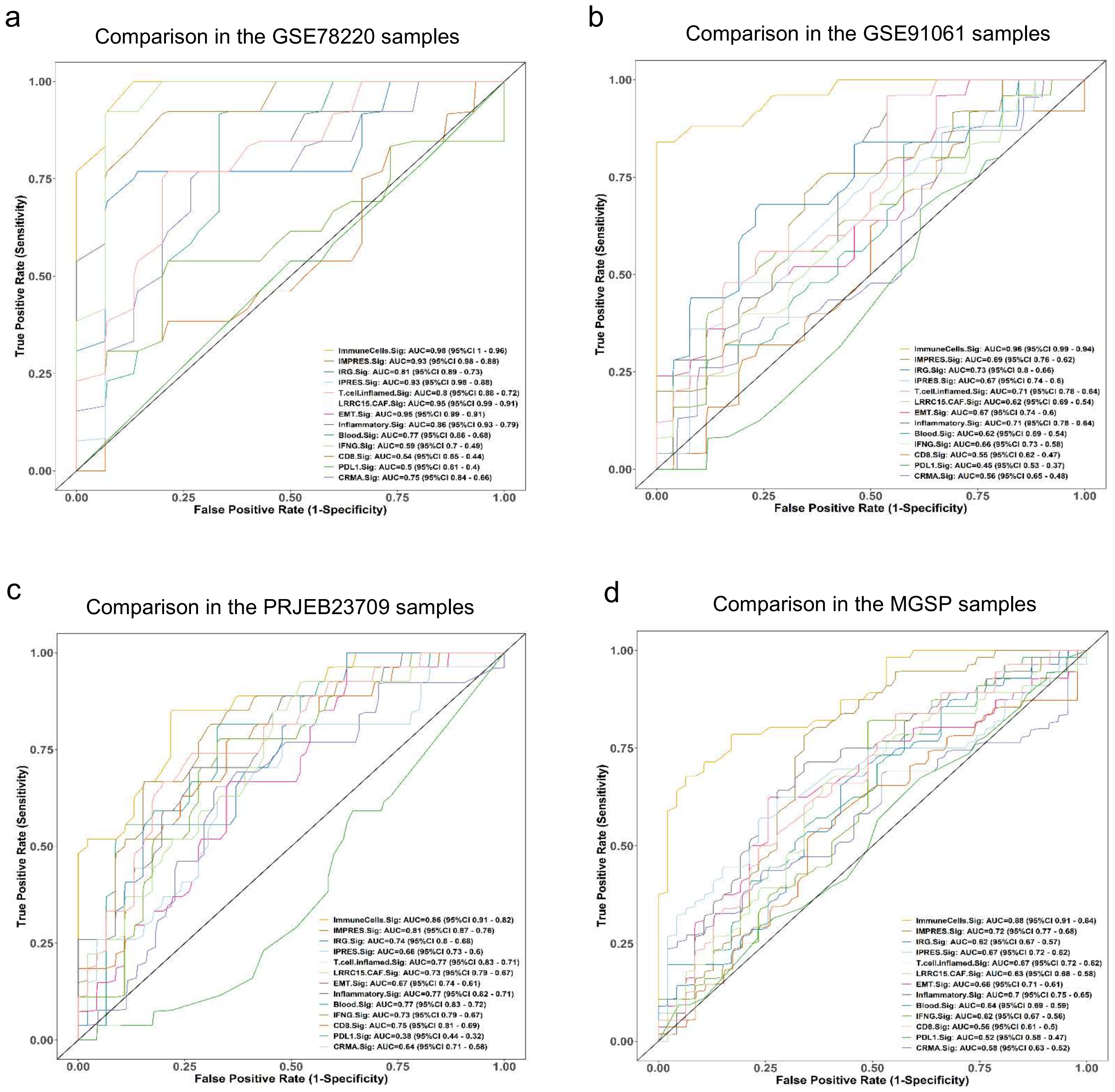

Supplementary Figure 12. Comparison of the performance of ImmuneCells.Sig with other ICT response signatures. The multiple ROC (receiver operating characteristic) curves are shown for the 13 ICT response signatures in (a) for the GSE78220 dataset; (b) for the GSE91061 dataset; (c) for the PRJEB23709 dataset and (d) for the MGSP dataset.

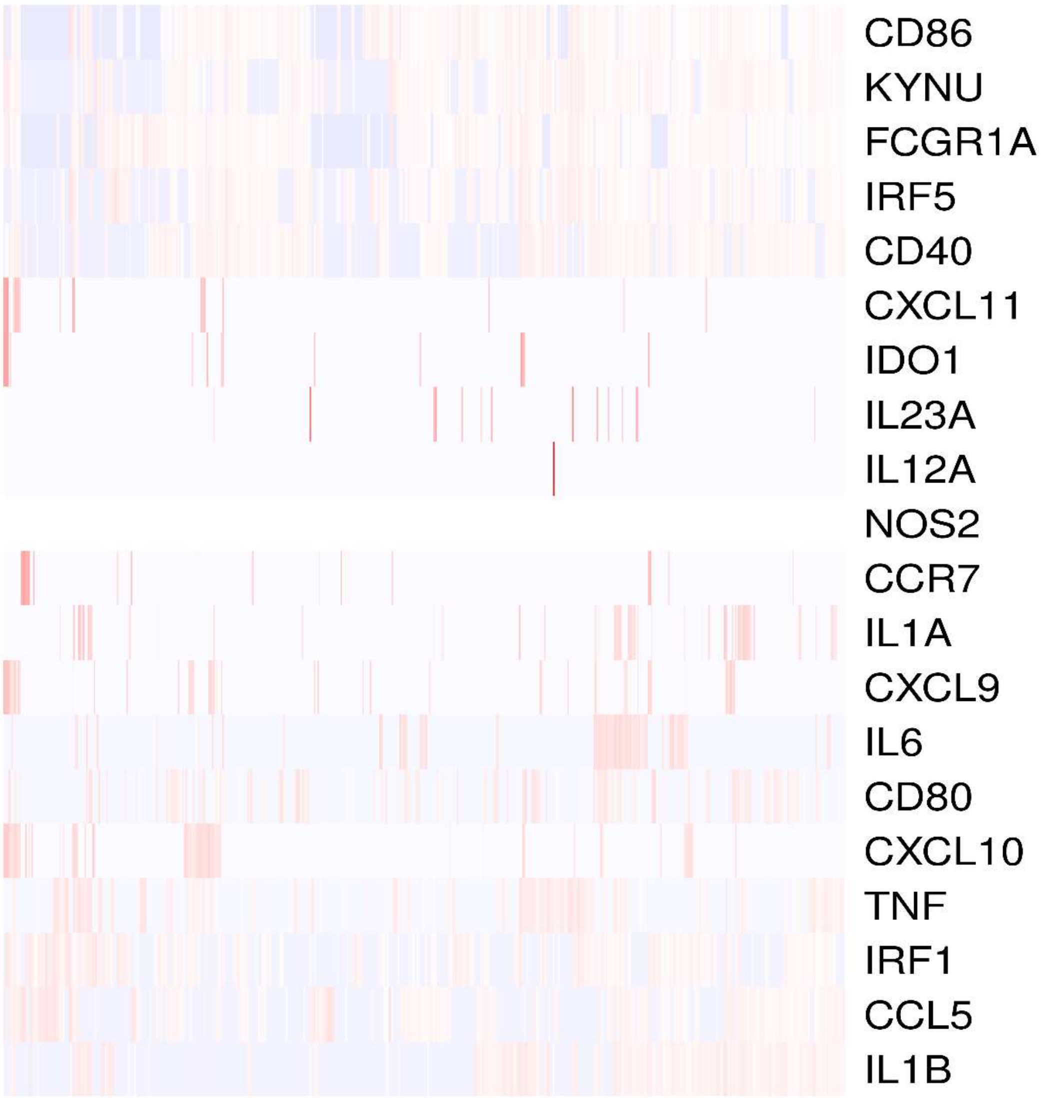

Supplementary Figure 13. The expression of M1 macrophage marker genes in the TREM2^{hi} population cells. The scRNA-seq dataset - GSE120575 was used in this analysis.

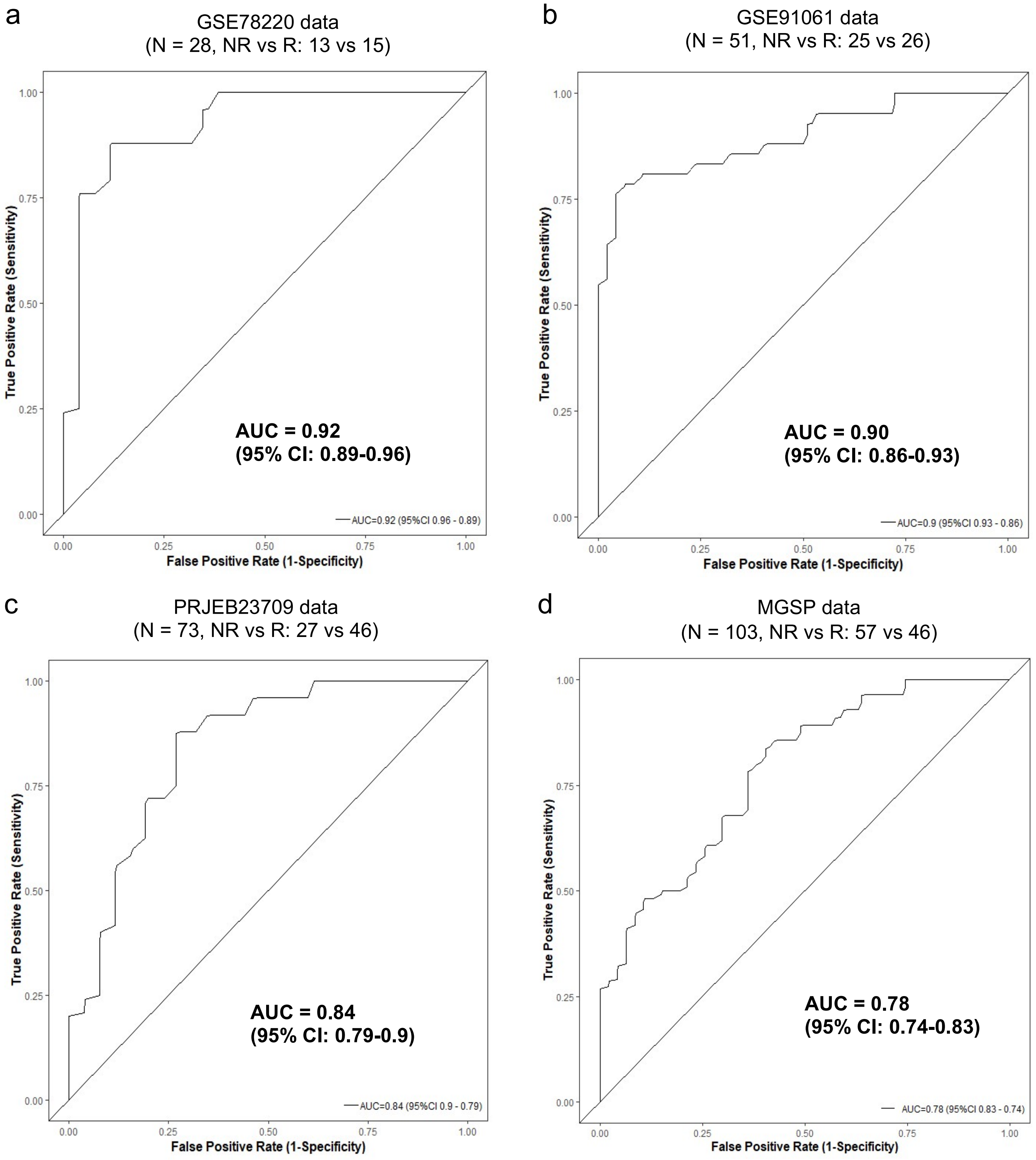

Supplementary Figure 14. Performance of a signature of immune cells. Using the gene signature of the three component cell clusters identified from single-cell data (TREM2^{hi} macrophages, Tgd_c21 γδ T cells and B_c22 B cells) to perform ICT outcome prediction analyses. The AUC values from this signature were 0.92, 0.90, 0.84 and 0.78 for the datasets of (a) GSE78220, (b) GSE91061, (c) PRJEB23709, and (d) MGSP, respectively.

Cluster	Immune cell population	Cell number	R_Percentage (% of CD45+)	NR_Percentage (% of CD45+)	NRvsR_Fold Difference
1	$CD8^+$ T cells	2502	11.45	17.39	1.5
2	Regulatory T cells	1886	12.94	10.87	-1.2
3	$CD4^+$ T cells	1521	11.99	7.96	-1.5
4	$CD8^+$ T cells	990	7.80	5.18	-1.5
5	$CD8^+$ T cells	926	6.00	5.52	-1.1
6	Macrophages/Monocytes	931	2.97	7.14	2.4
7	$CD8^+$ T cells	867	5.84	5.05	-1.2
8	γδ T cells	781	3.97	5.22	1.3
9	MKI67hi Lymph.	753	2.37	5.79	2.4
10	$CD8^+$ T cells	658	3.61	4.26	1.2
11	$CD8^+$ T cells	650	3.77	4.10	1.1
12	Macrophages/Monocytes	542	0.32	4.88	15.1
13	B cells	520	7.03	1.20	-5.8
14	B cells	519	5.63	1.92	-2.9
15	NK cells	490	3.15	2.94	-1.1
16	MKI67hi Lymph.	357	1.69	2.45	1.5
17	B cells	330	4.03	0.99	-4.1
18	Plasma cells	309	2.07	1.81	-1.1
19	Dendritic cells	274	0.84	2.12	2.5
20	$CD8^+$ T cells	202	0.59	1.58	2.7
21	γδ T cells	146	0.11	1.31	12.1
22	B cells	99	1.47	0.16	-9.3
23	Macrophages/Monocytes	38	0.36	0.17	-2.1

Supplementary Table 1. The percentages of the cluster-specific cells of the $CD45^+$ immune cells for each group (Responder - 'R'; Non-Responder - 'NR'). The scRNA-seq dataset - GSE120575 was used in this analysis.

Non-responders	Sample group	Sample number	Single-cell number
G1	NR-before-anti-PD-1	8	2604
G2	NR-before-anti-CTLA4	1	288
G3	NR-before-anti-CTLA4+PD-1	1	311
G4	NR-after-anti-PD-1	18	6334
G5	NR-after-anti-CTLA4	0	0
G6	NR-after-anti-CTLA4+PD-1	3	1190
Responders	Sample group	Sample number	Single cell number
G7	R-before-anti-PD-1	4	1191
G8	R-before-anti-CTLA4	1	229
G9	R-before-anti-CTLA4+PD-1	4	1305
G10	R-after-anti-PD-1	5	1524
G11	R-after-anti-CTLA4	0	0
G12	R-after-anti-CTLA4+PD-1	3	1315

Supplementary Table 2. Melanoma sample number and single cell number of stratified groups. The scRNA-seq dataset - GSE120575 was used in this analysis.
