# Supplementary material for: A gene expression signature of TREM2hi macrophages and γδ T cells predicts immunotherapy response
Source: Nat Commun. 2020 Oct 8;11:5084. doi: 10.1038/s41467-020-18546-x (PMC7545100; doi:10.1038/s41467-020-18546-x)
Supplement: Supplementary file 3 — Descriptions of Additional Supplementary Files [file 41467_2020_18546_MOESM3_ESM.pdf]

**Descriptions of Additional Supplementary Files**

**Supplementary Data 1.**

**Description:** Single-cell differential expression (DE) analyses results of cluster 6 macrophages (Inflammatory macrophages).

**Supplementary Data 2.**

**Description:** Single-cell differential expression (DE) analyses results of cluster 12 macrophages (TREM2hi macrophages).

**Supplementary Data 3.**

**Description**: Single-cell differential expression (DE) analyses results of cluster 23 macrophages (Immunoregulatory related macrophages).

**Supplementary Data 4.**

**Description**: Reactome pathways analysis for cluster 6 macrophages (IDO1hi inflammatory macrophages) specific genes detected by Seurat.

**Supplementary Data 5.**

**Description:** Reactome pathways analysis for cluster 12 macrophages (TREM2hi macrophages) specific genes detected by Seurat.

**Supplementary Data 6.**

**Description:** Reactome pathways analysis for cluster 23 macrophages (Immunoregulatory related macrophages) specific genes detected by Seurat.

**Supplementary Data 7.**

**Description:** Single-cell differential expression analyses results of the comparison of Tgd_c21 to Tgd_c8 cells.

**Supplementary Data 8.**

**Description:** Single-cell differential expression analyses results of the comparison of B_c22 to other B cell clusters.

**Supplementary Data 9.**

**Description:** Detailed information of the genes of ICT outcome signature - ImmuneCells.Sig.
