# Supplementary material for: A gene expression signature of TREM2hi macrophages and γδ T cells predicts immunotherapy response
Source: Nat Commun. 2020 Oct 8;11:5084. doi: 10.1038/s41467-020-18546-x (PMC7545100; doi:10.1038/s41467-020-18546-x)
Supplement: Supplementary file 13 — Reporting Summary [file 41467_2020_18546_MOESM13_ESM.pdf]

nature research

Corresponding author(s): Ming You
Last updated by author(s): Aug 14, 2020

Reporting Summary

Nature Research wishes to improve the reproducibility of the work that we publish. This form provides structure for consistency and transparency in reporting. For further information on Nature Research policies, see our Editorial Policies and the Editorial Policy Checklist.

Statistics

For all statistical analyses, confirm that the following items are present in the figure legend, table legend, main text, or Methods section.

n/a	Confirmed	
[ ]	[x]	The exact sample size (*n*) for each experimental group/condition, given as a discrete number and unit of measurement
[ ]	[x]	A statement on whether measurements were taken from distinct samples or whether the same sample was measured repeatedly
[ ]	[x]	The statistical test(s) used AND whether they are one- or two-sided<br>*Only common tests should be described solely by name; describe more complex techniques in the Methods section.*
[x]	[ ]	A description of all covariates tested
[x]	[ ]	A description of any assumptions or corrections, such as tests of normality and adjustment for multiple comparisons
[x]	[ ]	A full description of the statistical parameters including central tendency (e.g. means) or other basic estimates (e.g. regression coefficient) AND variation (e.g. standard deviation) or associated estimates of uncertainty (e.g. confidence intervals)
[ ]	[x]	For null hypothesis testing, the test statistic (e.g. *F*, *t*, *r*) with confidence intervals, effect sizes, degrees of freedom and *P* value noted<br>*Give P values as exact values whenever suitable.*
[x]	[ ]	For Bayesian analysis, information on the choice of priors and Markov chain Monte Carlo settings
[x]	[ ]	For hierarchical and complex designs, identification of the appropriate level for tests and full reporting of outcomes
[x]	[ ]	Estimates of effect sizes (e.g. Cohen's *d*, Pearson's *r*), indicating how they were calculated

Our web collection on statistics for biologists contains articles on many of the points above.

Software and code

Policy information about availability of computer code

Data collection	No software was used in data collection. All data were downloaded directly from the public databases.
Data analysis	R software, version 3.5.3 (R Project for Statistical Computing); Seurat v3.0; Bowtie-TopHat (version 2.0.4); HTSeq v0.11.1; DESeq2 v1.28.1; Ingenuity Pathway Analysis software (IPA release June 2020); Gene Set Variation Analysis (GSVA v1.36.2); Gene Set Enrichment Analysis (GSEA v4.0.0); Fast Gene Set Enrichment Analysis (fgsea v1.15.1); Protein ANalysis THrough Evolutionary Relationships (PANTHER v15.0); Gene Ontology database Released 2019-07-03; The cancerclass v1.32.0 R package.

For manuscripts utilizing custom algorithms or software that are central to the research but not yet described in published literature, software must be made available to editors and reviewers. We strongly encourage code deposition in a community repository (e.g. GitHub). See the Nature Research guidelines for submitting code & software for further information.

Data

Policy information about availability of data

All manuscripts must include a data availability statement. This statement should provide the following information, where applicable:

- Accession codes, unique identifiers, or web links for publicly available datasets
- A list of figures that have associated raw data
- A description of any restrictions on data availability

All of the data in this study have been obtained from publicly available sources. Gene Ontology database Released 2019-07-03 [http://geneontology.org/] was used in pathway analyses. The first set of scRNA-seq data were retrieved from Gene Expression Omnibus (GEO) under accession number GSE120575 [https://www.ncbi.nlm.nih.gov/geo/query/acc.cgi?acc=GSE120575]. For the validation purposes, the other two scRNA-seq data of melanoma and BCC were retrieved under accession numbers GSE115978 [https://www.ncbi.nlm.nih.gov/geo/query/acc.cgi?acc=GSE115978] and GSE123813 [https://www.ncbi.nlm.nih.gov/geo/query/acc.cgi?acc=GSE123813]. For the development of the ICT outcome signature, we analyzed the transcriptome-level gene expression data set of an immune

checkpoint therapy (ICT) study. The corresponding gene expression data were retrieved under accession number GSE78220 [https://www.ncbi.nlm.nih.gov/geo/query/acc.cgi?acc=GSE78220]. For the validation of the identified ICT outcome signature - ImmuneCells.Sig, we analyzed three additional large public gene expression datasets of immunotherapy. The first dataset was retrieved under accession number GSE91061 [https://www.ncbi.nlm.nih.gov/geo/query/acc.cgi?acc=GSE91061]. The second dataset PRJEB2370922 was retrieved from the corresponding website [https://www.ebi.ac.uk/ena/data/view/PRJEB23709]. We used the RNA-seq data of the 73 pre-treatment tumors from the PRJEB23709 study. Among these 73 samples, 41 are from the melanoma patients subjected to anti-PD-1 therapy and consist of 19 non-responders and 22 responders; 32 are from the melanoma patients subjected to combined anti-PD-1 and anti-CTLA-4 therapy and consist of 8 non-responders and 24 responders. The third dataset is from a large melanoma genome sequencing project23, from which the whole-transcriptome sequencing (RNA-seq) data from 103 pretreatment tumor tissue samples from 103 patients with distinct ICT outcomes (47 responders and 56 non-responders) were available and used for validation in this study. This dataset was named as MGSP (melanoma genome sequencing project) and available in dbGaP under accession number phs000452.v3.p1 [https://www.ncbi.nlm.nih.gov/projects/gap/cgi-bin/study.cgi?study_id=phs000452.v3.p1]. The source data used in the scRNA-seq and gene signature analysis are also available at https://github.com/donghaixiong/Immune_cells_analysis.

Field-specific reporting

Please select the one below that is the best fit for your research. If you are not sure, read the appropriate sections before making your selection.

[x] Life sciences   [ ] Behavioural & social sciences   [ ] Ecological, evolutionary & environmental sciences

For a reference copy of the document with all sections, see nature.com/documents/nr-reporting-summary-flat.pdf

Life sciences study design

All studies must disclose on these points even when the disclosure is negative.

Sample size	This study is based on the analyses of the publicly available datasets. In total, three scRNA-seq datasets from 90 samples (48+31+11) and four bulk RNA-seq datasets from 255 samples (28+51+73+103) of cancer patients subjected to immune checkpoint therapy were analyzed. The numbers are sufficient because prior single-cell studies in cancer immunotherapy used at most two or three datasets for validation with sample sizes far less than our sample size for validation that is 297 samples from 6 studies in addition to the initial set of 48 samples. For example, the representative study by Jerby-Arnon et al. [Cell 175, 984-997 e924 (2018)] had used 2 validation cohorts with a total number of 138 samples. Therefore, the sample size in our study is sufficient to analyze meaningful differences in the available cohorts.
Data exclusions	This study is based on the analyses of the publicly available datasets. No data in those previous studies were excluded.
Replication	No replication possible as study used clinical trial samples.
Randomization	Our study is based on the 7 component immune checkpoint therapy studies previously published. In those studies, patients were recruited to the associated clinical trial of immune checkpoint therapy and no randomization was used for patient samples, since all patients received immune checkpoint therapy, and no control (no therapy) samples were analyzed in the studies.
Blinding	No blinding was used for patient identity, since all patients received immune checkpoint therapy, and no control (no therapy) samples were analyzed in this study.

Reporting for specific materials, systems and methods

We require information from authors about some types of materials, experimental systems and methods used in many studies. Here, indicate whether each material, system or method listed is relevant to your study. If you are not sure if a list item applies to your research, read the appropriate section before selecting a response.

Materials & experimental systems

n/a	Involved in the study	
[x]	[ ]	Antibodies
[x]	[ ]	Eukaryotic cell lines
[x]	[ ]	Palaeontology and archaeology
[x]	[ ]	Animals and other organisms
[x]	[ ]	Human research participants
[x]	[ ]	Clinical data
[x]	[ ]	Dual use research of concern

Methods

n/a	Involved in the study	
[x]	[ ]	ChIP-seq
[x]	[ ]	Flow cytometry
[x]	[ ]	MRI-based neuroimaging
